# Atrazine Exposure and Reproductive Dysfunction through the Hypothalamus-Pituitary-Gonadal (HPG) Axis

**DOI:** 10.3390/toxics3040414

**Published:** 2015-11-02

**Authors:** Sara E. Wirbisky, Jennifer L. Freeman

**Affiliations:** School of Health Sciences, Purdue University, 550 Stadium Mall Drive, West Lafayette, IN 47907, USA; E-Mail: swirbisk@purdue.edu

**Keywords:** atrazine, endocrine disrupting chemical, hormones, hypothalamus-pituitary-gonadal axis, reproductive dysfunction

## Abstract

Endocrine disrupting chemicals (EDC) are exogenous agents that alter endogenous hormone signaling pathways. These chemicals target the neuroendocrine system which is composed of organs throughout the body that work alongside the central nervous system to regulate biological processes. Of primary importance is the hypothalamic-pituitary-gonadal (HPG) axis which is vital for maintaining proper reproductive function. Atrazine (2-chloro-4-ethylamino-6-isopropylamino-1,3,5-triazine) is a pre-emergent herbicide used to prevent the growth of weeds on various crops. This herbicide is reported to widely contaminate potable water supplies everywhere it is applied. As such, the European Union banned the use of atrazine in 2004. Currently the United States Environmental Protection Agency regulates atrazine at 3 parts per billion (ppb; μg/L) in drinking water, while the World Health Organization recently changed their drinking water guideline to 100 ppb. Atrazine is implicated to be an EDC that alters reproductive dysfunction by targeting the HPG axis. However, questions remain as to the human health risks associated with atrazine exposure with studies reporting mixed results on the ability of atrazine to alter the HPG axis. In this review, the current findings for atrazine’s effects on the HPG axis are examined in mammalian, anuran, and fish models and in epidemiological studies.

## 1. Introduction

The endocrine system is comprised of numerous organs throughout the body that work in tandem with the central nervous system (CNS) to regulate biological processes. Of primary importance is the reproductive system which is regulated by the hypothalamic-pituitary-gonadal (HPG) axis. Briefly, the HPG axis begins in the hypothalamus which serves as the integration center through the release of gonadotropin releasing hormone (GnRH) into the hypophyseal portal system which stimulates the synthesis and secretion of the gonadotropins (luteinizing hormone (LH) and follicle stimulating hormone (FSH)) from the anterior pituitary gland [1]. In females, LH exerts its function on the ovaries to promote ovulation and develop the corpus luteum; while FSH promotes follicular maturation and the synthesis of ovarian estrogens [2]. In males, LH acts on Leydig cells to promote the synthesis and secretion of testosterone, while FSH binds to Sertoli cells in order to promote spermatogenesis [3]. Additional hormones including activin, inhibin, and follistatin help maintain the positive and negative feedback systems required for gonadotropin homeostasis. Lastly, estradiol, progesterone, and testosterone produced by the ovaries and testes regulate the positive and negative feedback loops for proper reproductive function. A more in depth explanation of the HPG axis can be found in Vadakkadath and Atwood [4].

Endocrine disrupting chemicals (EDCs) are exogenous agents that alter endogenous hormone signaling pathways. Due to rapid industrialization, the production of these chemicals and their release into the environment has grown dramatically. Therefore, public concern about the effects of EDCs on human health has also increased substantially and heightened the need for further research into the mechanistic effects of these compounds [5,6]. EDCs are diverse in structure and are present in many products such as plasticizers, pharmaceuticals, and pesticides, allowing for a wide range of human exposures. Epidemiological studies have focused on the association between EDC exposure and various adverse health states, diseases, and disorders including reproductive dysfunction [7,8,9,10]. Although progress is being made, further work is needed to understand the mechanisms behind the adverse health outcomes associated with EDC exposure [6].

Uncovering the mechanisms of action behind the effects of EDCs on the reproductive system is of key interest as reproduction and embryonic development are highly susceptible biological processes [11]. Moreover, evidence suggests that exposure to EDCs can cause adverse effects not only in organisms that come into contact with them, but also to future progeny of exposed individuals [12,13,14]. Research investigating the mechanisms behind EDCs has focused on how EDCs interact with hormone receptors of the nuclear receptor (NR) family. In mammals, NRs are involved in numerous biological functions including fetal development, homeostasis, reproduction, metabolism, and response to xenobiotic substances [6]. Primary hormone receptors that belong to this family are the estrogen receptors (ER), androgen receptors (AR), and progesterone receptors (PR). However, thyroid hormone receptors (TRs), retinoid X receptors (RXR), and peroxisome-proliferator-activated receptors (PPAR) are also coming into the forefront. EDCs have the capability to directly bind to these receptors, therefore eliciting agonistic or antagonistic effects. Some examples of EDCs that target NRs include dichlorodiphenyltrichloroethane (DDT), polychlorinated biphenyls (PCBs), bisphenol A (BPA), polybrominated biphenyls (PBB), and polycyclic aromatic hydrocarbons (PAHs).

Numerous challenges are identified and need to be overcome when aiming to understand the mechanisms of action of EDCs. First, is the variable persistence in the body and in the environment which can range from a few days (e.g., bisphenol A (BPA)) to years (e.g., 1,1-dichloro-2,2-*bis*(*p*-chlorophenyl) ethylene (DDE)) [15]. This variation in persistence can make linking EDC exposure to adverse health outcomes a challenge. Additionally, the exposure period and duration can lead to significant adverse effects specifically during the critical *in utero* or developmental periods [5]. Further complicating our understanding of how EDCs disrupt hormonal homeostasis is their lack of a traditional dose response, often exhibiting a non-monotonic dose curve which provides a challenge in defining threshold levels. As such, during the 1990s EDCs became part of the “low dose hypothesis” indicating that these compounds can have adverse effects at or below environmentally allowable levels especially on reproduction and development [16].

Atrazine (2-chloro-4-ethylamino-6-isopropylamino-1,3,5-triazine) is a pre-emergent herbicide used to prevent the growth of broadleaf and grassy weeds on crops such as corn, sorghum grass, sugar cane, and wheat [17,18,19,20] that is reported to have endocrine disrupting effects. The United States Environmental Protection Agency (U.S. EPA) estimates approximately 76.5 million pounds of atrazine are applied annually in the United States, making it one of the most widely used herbicides [21]. The U.S. EPA has set the maximum contaminant level (MCL) for atrazine at 3 μg/L in drinking water supplies; however, during spring and summer months, this level is often exceeded [17,18,22,23]. Due to the high solubility, mobility in soil, long half-life, and widespread water contamination, atrazine was banned in the European Union in 2004. Prior to this, Italy and Germany banned the use of atrazine in 1991 with Sweden, Finland, and Denmark following suit in 1994 [24,25]. Alternatively, the World Health Organization (WHO) recently revised the allowable level of atrazine in drinking water to 100 μg/L [26]. Today, atrazine is still in use in more than 70 countries including Argentina, Brazil, China, and Mexico [25,27].

This article seeks to provide a comprehensive review of the new insights into atrazine exposure over the last twenty years with a focus on providing a thorough investigation of reproductive dysfunction elicited by atrazine exposure through the HPG axis in mammalian, anuran, and fish models and in epidemiological studies. An online search through the NCBI database based upon atrazine exposure and the HPG axis was assessed from 1995 through July 2015. Literature investigating the effects of atrazine on the hypothalamus-pituitary-adrenal (HPA) and hypothalamus-pituitary-thyroid (HPT) axes, and neurotransmitter studies were excluded.

## 2. The Effects of Atrazine on Female Reproductive Function in Mammalian Models

The primary focus of the endocrine disrupting properties of atrazine on the female HPG axis has been at the forefront of investigation as reproduction and embryonic development are highly susceptible to chemical insult. In the sections below, studies investigating the adverse effects of atrazine at various life stages are discussed followed by the genetic and upstream cellular mechanisms behind the observed morphological alterations in various female mammalian models (Table 1).

**Table 1:** Reproductive effects of atrazine in female mammalian models.

Reference	Species	Exposure	Duration	Outcomes
***In vitro***				
Pogrmic-Majkic *et al.* [28]	Rat granulosa cells	0 or 20 μM	48 h	Increase in progesterone; Increase in progesterone/estradiol ratio; Increase in *Star* and *Cyp11a1*; Activation of AKT and CREB
Fa *et al.* [29]	Rat granulosa cells	0, 10, or 20 μM	12 or 48 h	Increase in estradiol in 8-Br-cAMP stimulated granulosa cells; Decrease in *Cyp11a1* and *Lhr* expression; Decrease in ovulatory gene expression (*Areg, Ereg, PgR*)
Basini *et al.* [30]	Swine granulosa cells	0, 0.1, or 10 μM	48 h	Decrease in estradiol at 0.1 μM; Increase in progesterone at 10 μM; Increase in VEGF at 0.1 and 10 μM; Increase in NO at 10 μM
Tinfo *et al.* [31]	Granulosa cells and H295R adrenal cortical carcinoma cells	0 or 10 μM	24 h	Increased estradiol and aromatase activity after 24 h in granulosa cells; Increased estradiol and estrone in H295R cells; Increase in progesterone in both cell types
Holloway *et al.* [32]	Human granulosa and endometrial stromal cells	0, 1 nM, or 1 μM	24 h	Increase in aromatase activity in granulosa cell cultures; No alterations in aromatase protein or proportion of cells expressing aromatase
Sanderson *et al.* [33]	H295R adrenal cortical carcinoma cells	0–30 μM	24 h	Increase in CYP19A1 activity; Increase in *CYP19* mRNA
**Gestational**				
Davis *et al.* [34]	Sprague-Dawley rats	0, 1, 5, 20, or 100 mg/kg/day	GD 14–21	Delay in vaginal opening at 100 mg/kg/day; No alterations in mammary gland development on PND 45; No alterations in estrous cyclicity through PND 272
Hovey *et al.* [35]	Long-Evans rats	0, 6.5, 50, or 100 mg/kg/day	GD 13–19	No effects on mammary gland development beyond a transitory response to high doses at PND1
Rayner *et al.* [36]	Long-Evans rats	0 or 100 mg/kg/day	GD 13–15 or 15–17 or 17–19 or 13–19	Delay in vaginal opening in the GD 13–15 treatment group; No alterations in estrous cyclicity from PND 37–67; Decrease in area of mammary gland as soon as PND 4; Delays in development of mammary glands through PND 67; No alterations in serum hormone levels
Rayner *et al.* [37]	Long-Evans rats	0 or 100 mg/kg/day	GD 15–19	Delay in age vaginal opening; Delays in mammary gland epithelial development; No alterations in uterine or ovarian weight; No alterations in estrous cyclicity or serum hormone levels
**Peripubertal**				
Ashby *et al.* [38]	AP and Sprague-Dawley rats	0, 10, 30, or 100 mg/kg/day	PND 21 up to PND 46	Decrease in uterine weight at 100 mg/kg/day at PND 30 and PND 33; Delay in age of vaginal opening
Laws *et al.* [39]	Wistar rats	0, 12.5, 25, 50, 100, or 200 mg/kg/day	PND 22- PND 41	Delay in VO in the 50, 100, and 200 mg/kg/day treatment groups; Decreases in pituitary, ovary, uterine, adrenal, and kidney weight in 200 mg/kg/day at PND 41; No alterations in T_3_, T_4_, or TSH; Alterations in estrous cycle in first 15 days following VO at 100 mg/kg/day; No alterations in estrous cyclicity from PND 57–149
**Adult**				
Foradori *et al.* [40]	Sprague Dawley and Long-Evans rats	0.75–100 mg/kg/day oral gavage: 300–1460 ppm diet	4 days	Decrease in LH surge and area under the curve in Sprague-Dawley rats treated by oral gavage; Decrease in mean number of corpora lutea and number of ova in Sprague-Dawley rats treated by oral gavage; No change with dietary consumption
Goldman *et al.* [41]	Long Evans rats	0, 10, 30, or 100 mg/kg/day	1 or 4 days	Decrease in LH surge; Increase in serum progesterone; Increase in *Kiss1* mRNA expression
Foradori *et al.* [42]	Wistar rats	0, 50, 100, or 200 mg/kg/day	4 days	No alterations in gene expression, peptide levels, or immunoreactivity; Reduction observed in GnRH pulse frequency; Increase in GnRH pulse amplitude
Quignot *et al.* [43]	Sprague-Dawley rats	0 or 200 mg/kg/day	14 days	Decrease in uterine and ovary weight; Increase in estrone and estradiol; Increase in ovarian aromatase expression; Increase in estrous cycle; Alterations in steroidogenic genes
Taketa *et al.* [44]	Sprague-Dawley rats	0 or 300 mg/kg/day	4 days or 2 weeks	Abnormal estrus cycle (persistent diestrus); Luteal cell hypertrophy and atretic follicles observed; Increase in serum progesterone; Increase in steroidogenic gene *SR-B1*
Foradori *et al.* [45]	Wistar rats	0, 50, 100, or 200 mg/kg/day	4 days	Reduction in magnitude of LH and FSH surges; Decrease in GnRH neurons; Measures of HPG activation return to normal 4 days after cessation
Foradori *et al.* [46]	Wistar rats	0, 50, 100, or 200 mg/kg/day	4 days	Decrease in LH pulse frequency and increase in pulse period and pulse amplitude in 200 mg/kg/day treatment group
Shibayama *et al.* [47]	Sprague-Dawley rats	0, 3, 30, or 300 mg/kg/day	2 or 4 weeks	Loss of corpora lutea; Increase in atretic follicles; Swelling of leuteal cells; Prolonged estrous cycle; Decrease in ovarian and uterine weight
Juliani *et al.* [48]	Wistar rats	0, 0.75, or 400 mg/kg/day	14 (0.75 mg/kg) or 30 days (400 mg/kg)	No increases in atretic antral follicles, but intensity level of apoptosis in granulosa cells was visibly higher than control in the 0.75 mg/kg/day group; Disorganized granulosa cells, discontinuous zona pellucida, high intensity of apoptosis in atretic antral follicles in the 400 mg/kg/day treatment group
McMullin *et al.* [49]	Sprague-Dawley rats	0, 30, 100, or 300 mg/kg/day	5 days	Decrease in LH at 30, 100, and 300 mg/kg/day; No binding to estrogen receptor *in vivo*
Cooper *et al.* [50]	Sprague-Dawley and Long-Evans rats	0, 50, 100, 200, or 300 mg/kg/day	1, 3, or 21 days	Suppression of serum LH and PRL; Increase in pituitary LH

Abbreviations: AKT = Protein kinase B; CREB = cAMP response element-binding protein; FSH = Follicle stimulating hormone; GD = Gestational Day; GnRH = Gonadotropin releasing hormone; HPG = Hypothalamus-pituitary-gonadal; LH = Luteinizing hormone; NO = Nitrous oxide; PND = Post-natal day; PRL = Prolactin; SR-B1 = Scavenger-receptor B1; TSH = Thyroid stimulating hormone; T_3_ = Triiodothyronine; T_4_ = Tetraiodothyronine; VEGF = vascular endothelial growth factor; VO = Vaginal opening.

### 2.1. Gestational Atrazine Exposure in Females

Studies examining the gestational effects of atrazine on female offspring have primarily focused around mammary gland development and time of vaginal opening (VO). A study conducted by Rayner *et al.*, treated pregnant Long-Evans rats with 100 mg/kg/day atrazine from gestational day (GD) 15–19. Once pups were born, cross-fostering was used to determine if observable effects were dam-mediated (gestational and lactational) or direct (gestational). Results indicated a significant delay in VO in the litters which also received milk from atrazine exposed dams. Furthermore, offspring in all treatment groups had delays in mammary epithelial development which was evident as early as postnatal day (PND) 4 and continued to PND 40. Conclusions drawn from this study were that the delay in VO was mediated through gestational and lactational atrazine exposure, while brief direct exposure *in utero* caused the delay in mammary gland development [37]. A follow-up study was performed in order to investigate whether the previously observed delay in mammary gland epithelium was dependent upon particular developmental stages. This study exposed pregnant Long-Evans rats to the same atrazine concentration (100 mg/kg/day); however, dosing periods ranged from GD 13–15, GD 15–17, GD 1–19, or GD 13–19. These studies confirmed the atrazine induced delay in VO in the GD 13–15 treatment group. Additionally, a delay in mammary gland development which began at PND 4 and continued through PND 64 was confirmed, with GD 17–19 being the most sensitive developmental window [36]. In contrast to the aforementioned studies, two studies were conducted which found no alterations in mammary gland epithelial development [34,35]. Discrepancies of these data sets have been hypothesized to be due to technical variations between the studies. Hovey *et al.*, excluded any whole mount tissue samples that did not contain the entire ductal network; whereas the assumption was made that the studies conducted by Rayner *et al.*, did not evaluate the entire ductal system; therefore, compromising a complete analysis [31,35,36,37]. Data from these studies provides evidence that atrazine may cause alterations in mammary gland epithelial development; however, rigorous quantitative analysis should be performed in future studies.

A controversial debate that circulates around atrazine is whether or not atrazine elicits mammary gland tumor development. One study stated that a lifetime atrazine exposure (24 months) of 400 mg/L caused an earlier onset of mammary gland tumor development in Sprague-Dawley (SD) rats [51]. Additional studies have been completed by Stevens *et al.*, in Sprague-Dawley rats further supporting that atrazine exposure increases the incidence of mammary gland tumor development [52,53]. However, studies investigating incidence of tumor formation in Fischer-344 rats, did not find an increase in mammary tumor formation [54,55]. A key biological difference causing this strain specific effect of atrazine exposure is that Fischer-344 rodents maintain a normal estrous cycle throughout a greater portion of its life cycle compared to Sprague-Dawley rats. Therefore, allowing reproductive senescence to occur later in life [56]. In addition, it is well known that mammary tumor formation in rodents is hormone dependent and is common among aging rats [57]. Moreover, it has also been recognized that estradiol and prolactin can promote tumor growth. Eldridge *et al.*, determined that atrazine does not have estrogenic activity, but can alter the estrus cycle and increase estradiol; therefore, providing a hormonal milieu conducive to tumor formation [58]. Data regarding mammary tumor formation due to atrazine exposure appears to be trending towards the hypothesis that the development of mammary tumors is a secondary outcome of the neuroendocrine alterations that arise from atrazine exposure.

### 2.2. Peripubertal Atrazine Exposure in Females

As compared to peripubertal male studies (discussed in Section 3.2) fewer studies have aimed to address the effects of peripubertal atrazine exposure on the HPG axis in females. Similarly to the gestational studies, interest surrounding age of VO has been assessed along with estrous cyclicity as a marker of successful reproductive function. A study conducted by Ashby *et al.*, found that an atrazine treatment of 100 mg/kg/day from PND 21 up to PND 45 caused a decrease in uterine weight at PND 30 and PND 33 along with an increase in age of VO [38]. Results from this study are in agreement with an earlier study which showed that the same atrazine treatment (100 mg/kg/day) caused a delay in VO due to exposure from PND 22–41. Further results from this study displayed altered estrous cyclicity during the first 15 day interval following VO; however, the observational period in this study lasted through PND 149 in which from PND 57–149, no alterations in estrous cyclicity were observed [39]. This extended observational period is beneficial as only observing within the treatment period (PND 22–41) does not allow for the naturally occurring irregularity in female estrous cycles that occurs at the onset of puberty [39].

### 2.3. Adulthood Atrazine Exposure in Females

As previously mentioned, numerous neural mechanisms control the functioning of the HPG axis. The key regulator of this pathway is the hypothalamus which provides the neural signals eliciting the release of GnRH to act on the pituitary for the release of LH and FSH. Disruption of this neuroendocrine pathway can lead to nonresponsive gonadotrophs and a loss of reproductive function and has been the target of investigation for multiple studies. It has been shown that atrazine and its metabolites are lipid soluble and can cross the blood brain barrier; therefore, allowing atrazine to interact with GnRH neurons [59]. A study conducted by Foradori *et al.*, exposed ovariectomized female Wistar rats to 0, 50, 100, or 200 mg/kg/day for four days. Results from this study indicated that atrazine exposure of 100 and 200 mg/kg/day reduced the numbers of activated GnRH neurons [45]. To complement this data, an additional study was conducted which investigated the effects of atrazine directly on GnRH pulsatile release, gene expression, and peptide levels [42]. Atrazine elicited a reduction in GnRH pulses with higher pulse amplitude, confirming previous results [45]. In addition, no significant alterations were observed in the expression of GnRH mRNA or in GnRH peptide levels. As previously discussed, GnRH regulates the release of LH and atrazine has been reported to decrease LH pulse frequency and increase pulse period and amplitude [40,46,49,50,57]. The results from Cooper *et al.*, and Foradori *et al.*, have also demonstrated that atrazine does not affect the sensitivity of the pituitary to GnRH [46,50]. These data combined with an additional study in which atrazine increased the concentration of GnRH in the median eminence further implies that atrazine exerts its effects at the level of the hypothalamus and not the pituitary [57]. The combined studies presented, contribute significant weight into the effects of atrazine on the HPG axis through GnRH, LH, and FSH due to their strong experimental methodology. Although progress has been made, it is still unknown whether atrazine directly affects GnRH neurons or if it acts on one of the numerous neurotransmitter systems that can affect GnRH neurons [60,61,62].

Another possible mechanism of the observed reduction in GnRH resides further upstream in the hypothalamus. Kisspeptin neurons are located in the hypothalamic anteroventral periventricular nucleus (AVPV) and directly innervate GnRH neurons; therefore, potentially attenuating the GnRH pulses leading to the downstream reduction in LH [41,63,64]. Kisspeptin neurons also contain progesterone and estrogen receptors, which could contribute to disruption in ovarian histology and reproductive dysfunction. Although no studies have addressed whether or not atrazine directly affects kisspeptin neurons, a study conducted by Goldman *et al.*, observed an increase in *Kiss1* mRNA following atrazine exposure in female Long-Evans rodents [41]. This increase was observed only one day following atrazine administration with no alterations present following a four day exposure. This increase is in combination with an observed increase in progesterone (discussed below), further supporting that immediate effects of atrazine exposure can alter additional upstream regulators of GnRH neurons.

Atrazine exposure has been shown to cause various histological alterations within ovarian tissue. A study conducted by Shibayama *et al.*, treated Sprague-Dawley rats with 0, 3, 30 or 300 mg/kg/day for two to four weeks. Results from this study revealed a loss of corpora lutea, an increase in atretic follicles, swelling of luteal cells, and a prolonged estrus cycle (hypothesized to be prolonged diestrus) in the 300 mg/kg atrazine treatment group [47]. Furthermore, female Wistar rats exposed to 0.75 or 400 mg/kg/day reported no alterations in atretic antral follicles, but a large amount of preantral follicles presented disorganized granulosa cells and/or a degenerating oocyte. Ovotoxicity was also observed in the antral follicles via high intensity of apoptosis in granulosa cells, nucleolus fragmentation of the oocyte, and irregularities of the Zona Pellucida [48]. The ovotoxicity generated by atrazine occurred at a relatively high dose of 400 mg/kg/day for 14 days. These data demonstrate that high doses can elicit infertility and impair folliculogenesis. These data are in agreement with Laws *et al*. who report that atrazine at levels of 200 mg/kg can alter estrus cyclicity [39]. One study conducted in female pigs reported cystic ovarian degeneration and an increase in the persistence of the corpus luteum following an atrazine exposure of 2 mg/kg in feed for 19 days [65]. This report is in agreement with rodent studies; however there was a limited sample size. It has been identified that damage to antral follicles may lead to changes in hormone levels and/or estrus cyclicity [66], all of which have been observed in various rodent studies. These combined results suggest ovotoxicity and anovulatory effects of atrazine.

Progesterone (P4) is a key hormone that is needed for proper reproductive function, primarily in early luteinization and is known to be secreted from both the ovaries and adrenal glands [41]. An increase in progesterone levels due to atrazine exposure has been observed in pre-ovulatory granulosa cells and H295R adrenal cortical carcinoma cells [31]. In addition, *in vivo* studies have reported increases in progesterone following atrazine administration [41,44,67]. To further strengthen the disruption of ovarian function, a study utilizing female pigs reported an increase in serum P4 and a decrease in estradiol before the onset of estrus following dietary atrazine exposure [65]. These studies suggest that this elevation of progesterone during the transitional period from the immature to the pre-ovulatory stage demonstrates that atrazine alters ovarian function [28]. An additional mechanism behind the observed increase in progesterone following atrazine exposure is the reported decrease in the expression of luteinizing hormone receptors (LHR) in granulosa cells (discussed in Section 2.5). This decrease in LHR can elicit a decrease in LH and in estradiol functions in the ovary including the development of the corpora lutea, size of ovaries, and estrus cyclicity. As such, a synergistic relationship exists between LH and P4; as P4 increases (and remains elevated) it can adversely affect negative feedback loops of the HPG axis causing a continual decrease in LH. Furthermore, research has shown that higher levels of P4 inhibit follicle development and can potentiate follicular atresia [68]. Therefore, the observed increase in P4 could be contributing to the continued inhibition of the pre-ovulatory surge of LH leading to the histological and reproductive alterations observed in females.

Estradiol and testosterone are also key steroid hormones involved in proper reproductive function. Levels of these hormones are not as abundantly studied in female rodent models as compared to male models (discussed in Section 3.3). A study conducted by Quignot *et al.*, treated Sprague-Dawley rats with 200 mg/kg/day atrazine for 14 days observed a decrease in ovarian testosterone; however, no significant alterations were observed in plasma or ovarian estradiol [43]. A second study also observed no alterations in serum estradiol following an atrazine exposure of up to 100 mg/kg/day in Long-Evans rodents [41]. *In vitro* studies utilizing swine granulosa cells exposed to 0.1 μM atrazine for 48 h displayed a decrease in estradiol, while no alterations occurred at 10 μM [30]. A second study conducted by Fa *et al.*, reported that atrazine inhibits estradiol production in FSH stimulated granulosa cells at 20 μM. While in contrast, cAMP stimulated cells display an increase in estradiol following the same atrazine exposure [29]. These differences could be attributed to cell type as well as treatment periods.

### 2.4. Cellular and Genetic Mechanisms of Reproductive Dysfunction in Females

The mechanism responsible for the conversion of testosterone to estrogen is aromatase (*CYP19A1*). The effects of atrazine on *CYP19A1* are controversial (as discussed below). *In vitro* literature utilizing granulosa cells and adrenal cortical carcinoma (H295R) cells show that atrazine exposure increases aromatase and aromatase activity [32,33]. This increase in activity should account for increases in estradiol; however, as previously discussed, an increase in estradiol is not always observed [30,41,43].

**Figure 1:**
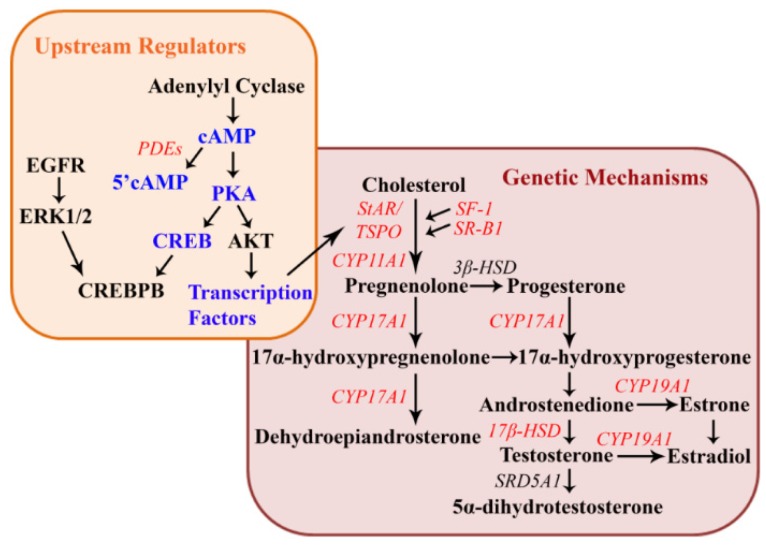
Working conceptual model of steroidogenesis and upstream regulators associated with atrazine exposure. This diagram represents the upstream cellular mechanisms (upstream regulators are in blue) and steroidogenic genes (in red) that are reported to be altered by atrazine exposure. All genes are included above as their human homologs.

Further mechanistic work was performed in order to understand the genetic components behind the observed gonadal histology and hormonal alterations. Rat granulosa cells treated with various concentrations of atrazine for 48 h found that atrazine exposure decreased luteinizing *hormone receptor* (*Lhr*) mRNA expression and the expression of ovulatory genes *amphiregulin* (*Areg)*, *epiregulin* (*Ereg*), and *progesterone receptor* (*PgR*) [29]. Decreased expression of the ovulatory genes could be a contributing factor to the increase in atretic follicles and cystic ovarian degeneration [47,48,59]. An upstream regulator of LH function is the ERK1/2 pathway [69]. Although atrazine does not directly affect the phosphorylation of ERK1/2, the activation of this pathway is hypothesized to be critical for the atrazine induced attenuation of *Lhr* mRNA expression [29]. A study conducted by Pogrmic-Majkic *et al.*, provided additional mechanistic data regarding the observed increases in abnormal ovarian morphology and progesterone elicited by atrazine exposure [31,41,44,47,48,59,67]. Findings suggest that atrazine causes an increase in P4, the P4/E2 ratio, and overexpression of luteal markers (*Star* and *Cyp11a1*) through the stimulation of the cAMP, AKT, ERK1/2, and CREBPB-signaling pathways most likely through the inhibition of *phosphodiesterase 4* (*Pde4*); therefore promoting premature luteinization in rat granulosa cells [28,33,70,71] (Figure 1).

### 2.5. Tissue Levels of Atrazine in Females

Few epidemiological studies have identified atrazine and/or its metabolites in the urine of agricultural workers. Therefore, the majority of epidemiological studies are retrospective in linking water concentrations of atrazine to reproductive dysfunction and birth defects (discussed in Section 5). Animal studies aiming to address the distribution of atrazine in tissues are limited. A study conducted by Fraites *et al.*, investigated the distribution of atrazine and its metabolites in maternal, fetal, and neonatal fluid and tissue samples following gestational and/or lactational exposure [72]. Pregnant Sprague-Dawley dams were exposed to 5 or 25 mg/kg/day from GD 14–20 or GD 18–20. The exposed dam had the highest levels of the atrazine metabolite diaminochlorotriazine (DACT) in the plasma, adrenal, brain, and mammary tissues. Atrazine contributed to less than 1% of total chlorotriazines identified in the plasma and brain. However, higher atrazine amounts were identified in the adrenal and mammary tissues. [72]. In addition, gonadal levels of atrazine and its metabolites were investigated. DACT was identifiable in ovarian tissue only at 25 mg/kg, while the 5 mg/kg treatment group reported levels of DACT below the limit of quantitation [72]. The strength of this study comes from the dose levels of 5 and 25 mg/kg, as the majority of rodent studies (as discussed in Section 2.1, Section 2.2 andSection 2.3) rely on higher levels (≥100 mg/kg). These lower levels allow for a more translatable approach to epidemiological studies (discussed in Section 5). A second study conducted by Quignot *et al.*, exposed female Sprague-Dawley rats to 200 mg/kg atrazine for 14 days and found an accumulation of atrazine in ovarian tissue [73]. Results from these studies provide support for the reproductive dysfunction elicited by atrazine exposure through multiple avenues. First, these studies clearly show that atrazine and DACT are present in brain tissue. Second, the presence of atrazine in mammary tissue supports key findings that show gestational and lactational exposure is detrimental to development [37]. In addition, the identification of atrazine in the adrenals provides support that atrazine can also target additional neuroendocrine pathways including the HPA axis. Although gonadal tissue requires intensive sample preparation due to their complex biological matrix and high lipid content, these studies show that atrazine is present in ovarian tissue [73] and highlight that further work is needed to link ovarian atrazine levels to observed histological, hormonal, and cellular alterations.

### 2.6. Conclusions

**Figure 2:**
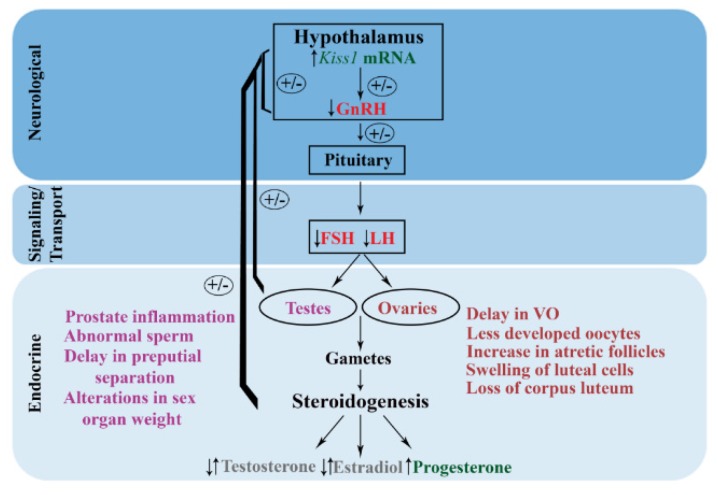
Working conceptual model of atrazine toxicity on the hypothalamus-pituitary-gonadal (HPG) axis across mammalian, anuran, and fish species. This diagram summarizes the main common alterations observed with atrazine exposure on the HPG axis based upon studies across multiple species and exposure paradigms. Atrazine exposure elicits an increase in *Kiss1* mRNA and a decrease in gonadotropin releasing hormone (GnRH) release leading to a downstream reduction in luteinizing hormone (LH) and follicle stimulating hormone (FSH) from the anterior pituitary. This reduction in gonadotropins elicits alterations in testosterone, estrogen, and progesterone. In addition, histological and morphological alterations in the ovaries and testes are observed and are ultimately dependent on time of exposure and dose. (Green indicates increases, pink indicates reductions, and grey indicates that both increases and reductions are reported. Histological and morphological effects in males are indicated in purple, while alterations in females are in red; VO: vaginal opening).

The effects of atrazine on the female mammalian HPG axis have shown numerous adverse impacts from *in vitro*, gestational, peripubertal, and adulthood exposures. These alterations ranged from delays in puberty, altered estrus cycles, atretic ovarian follicles, reduction in gonadotropins, and cellular and genetic alterations. These studies provide support of atrazine as an EDC and its ability to elicit reproductive dysfunction throughout multiple life stages (Figure 2). While questions still remain on human health relevancy of doses at which these adverse effects are observed, results from these studies begin to provide insight into the reproductive dysfunction observed in epidemiological studies.

## 3. The Effects of Atrazine on Male Reproductive Function in Mammalian Models

The primary focus of the endocrine disrupting effects of atrazine on the male reproductive system has progressed over the years but is still under investigation. In the sections below, studies investigating the adverse health outcomes at various life stages are discussed followed by the genetic and upstream cellular mechanisms behind the observed morphological alterations in various mammalian models (Table 2).

**Table 2:** Reproductive effects of atrazine in male mammalian models.

Reference	Species	Exposure	Duration	Outcomes
***In vitro***				
Forgacs *et al.* [74]	BLTK1 Murine Leydig cells	1, 3, 10, 30, 100, 300, or 600 μM	12, 24, or 48 h	Increase in progesterone and testosterone; Alterations in steroidogenic genes
Kucka *et al.* [70]	Sprague-Dawley anterior pituitary and Leydig cells	10–50 μM	Various time points	Increase in cAMP and prolactin in pituitary cells; Inhibition of PDE4 isoenzymes
Abarikwu *et al.* [75]	Wistar rat Leydig cells	Cytotoxic studies 0.5, 5, 10, 25, or 50 μg/mL	Cytotoxic studies 24–72 h; Gene expression studies 2 h	Decrease in cell viability at 48 and 72 h at 25 and 50 μM; Alterations in steroidogenic gene expression
**Gestational**				
Stanko *et al.* [76]	Long-Evans rats	100 mg/kg/day	GD 15–19	No body weight alterations; Delay in preputial separation; Increase in testosterone and decrease in estrone at PND 120; Decrease in estradiol at PND 180
Rosenberg *et al.* [77]	Sprague-Dawley rats	0, 10, 50, 75, or 100 mg/kg/day	GD14-Parturition	Increase in dead pups at 75 and 100 mg/kg; Increase in preputial separation at 100 mg/kg/day; Decrease in angiogenital distance at 100 mg/kg/day; No gross morphology alterations; Decrease in serum and intratesticular testosterone
**Peripubertal**				
Jin *et al.* [78]	ICR mice	50, 100, or 200 mg/kg/day	3 weeks	Decrease in body, testes, and liver weight; Decrease in testosterone; Increase in estradiol; Decrease in expression of steroidogenic genes
Pogrmic-Majkic *et al.* [79]	Wistar rats	*In vitro*: 1–50 μM *In vivo*: 200 mg/kg	24 or 72 h	Transient increases in steroidogenic genes after 24 h exposure followed by a decline
Pogrmic *et al.* [80]	Wistar rats	50 or 200 mg/kg/day	PND 23–50	Decrease in steroidogenic genes and androgens
Friedmann *et al.* [81]	Rat	50 mg/kg/day	PND 46–48 (acute); PND 22–48 (chronic)	Decrease in serum and intratesticular testosterone
Trentacoste *et al.* [82]	Sprague-Dawley rats	0, 100, or 200 mg/kg/day	PND 22–48	Delay in preputial separation; Decrease in serum LH and testosterone; Decrease in intratesticular testosterone; Decrease in organ weights
Stoker *et al.* [83]	Wistar rats	0, 12.5, 25, 50, 100,150, or 200 mg/kg	PND 23–53	Decrease in seminal vesicle and prostate weight; No alterations in testes weight; Delay in preputial separation; No alterations in serum testosterone at PND 53
**Adult**				
Song *et al.* [84]	Sprague-Dawley rats	38.5, 77, or 154 mg/kg/day	30 days	Decrease in spermatozoa number; Increase in spermatozoa abnormality; Decrease in serum testosterone; Decrease in expression of inhibin-B; Increase in FSH and LH
Jin *et al.*, [85]	ICR mice	0, 100, or 200 mg/kg/day	4 × 1 week	Decrease in testicular testosterone; Decrease in relative testes weight at 200 mg/kg; Decrease in steroidogenic gene expression
Riffle *et al.* [86]	Wistar rats	0, 5, 25, 75, 200 mg/kg/day	3 Days	Increase in serum progesterone in intact and castrated males at 25 and 200 mg/kg and 25, 75, and 200 mg/kg respectively; Increase in serum corticosterone at 25, 75, and 200 mg/kg in intact and castrated rats; Increase in testosterone and luteinizing hormone in intact males at 25 mg/kg; Alterations in proteins associated with testosterone production
Abarikwu *et al.* [87]	Wistar rats	0, 120, or 200 mg/kg/day	7 or 16 Days	Decrease in seminal vesicle and prostate weight at 200 mg/kg/day; Decreased sperm number with increased abnormal sperm at 120 and 200 mg/kg/day
Victor-Costa *et al.* [88]	Wistar rat	50, 200, or 300 mg/kg/day	7, 15, or 40 days	Decreased body weight, increased testes and adrenal weight; Decreased testosterone; Increased estradiol; Dilation of seminiferous tubules; Testicular atrophy
**Developmental Origins of Atrazine Toxicity**				
DeSesso *et al.* [89]	Wistar rats	0, 1, 5, 25, or 125 mg/kg/day	GD 6–21 or PND 2–21	No alterations in spermatid counts in testes, spermatozoa counts in epididymides, or plasma testosterone levels at PND 70 or PND 170; Increase in percentage of abnormal sperm on PND 70 at 125 mg/kg/day; PND exposure showed a reduction in absolute testes and epididymis weights in the 125 mg/kg/day treatment group on PND 70; No effect on plasma testosterone or sperm morphology at PND 70 and PND 170
Fraites *et al.* [72]	Sprague-Dawley rats	0, 1, 5, 20, or 100 mg/kg/day	GD 14–21	No alterations in testosterone production, timing of puberty, play behavior, AGD, or male sex organ weights at any atrazine treatment at PND 59
Rayner *et al.* [90]	Long-Evans rats	100 mg/kg/day	GD 15–19	Delay in preputial separation; Increase in lateral prostate weight at PND 120; No alterations in testes and seminal vesicle weight at PND 120 and PND 220; No alterations in serum testosterone and androstenedione, but a decrease in prolactin at PND 220

Abbreviations: AGD = Angiogenital distance; cAMP = 3′-5′-cyclic adenosine monophosphate; FSH = Follicle stimulating hormone; GD = Gestational day; LH = Luteinizing hormone; PDE4 = Phosphodiesterase 4; PND = Post-natal day.

### 3.1. Gestational Atrazine Exposure in Males

The first study of interest exposed pregnant Long-Evans rats to 100 mg/kg/day atrazine from GD 15–19 and male offspring were assessed at PND 120 and PND 180. Results demonstrated that gestational atrazine exposure caused a significant delay in preputial separation. In addition, no significant differences in testes weights, seminal vesicle weights, or alterations in prostate morphology were observed. Serum testosterone and estrone were increased at PND 120, while at PND 180 a decrease in estradiol and estrone was observed [76]. A second study exposed pregnant Sprague-Dawley rats to 100 mg/kg/day atrazine from GD14-parturition and found that atrazine exposure elicited an increase in the angiogenital distance (AGD) at PND 21 along with a delay in preputial separation. Further analysis at PND 60 found no alterations in body, testes, seminal vesicle, or prostate weight, while a reduction in serum and intratesticular testosterone was observed [77]. The results from these two studies are in agreement regarding an increase in age of preputial separation and no alterations in accessory organ weights; however, contradictory findings are present regarding the effect of atrazine on serum testosterone levels. It is known that preputial separation is androgen dependent and normally occurs when androgen levels are high. Results from Rosenberg *et al.*, support this hypothesis; however, findings of Stanko *et al.*, are contradictory as they have a delay in preputial separation along with an increase in testosterone. This could be due to the differences in assessment windows as there is a much longer latency period in the Stanko *et al.*, study [76,77].

### 3.2. Peripubertal Atrazine Exposure in Males

Peripubertal atrazine exposure in males has also been under investigation and poses fewer challenges than gestational exposures. One study exposing male juvenile Sprague-Dawley rats to 50 mg/kg/day of atrazine acutely from PND 46–48 or chronically from PND 22–48 showed a significant reduction in both serum and intratesticular levels of testosterone in both treatment groups [81]. In a similar study in which male Sprague-Dawley rats were exposed to 100 or 200 mg/kg/day atrazine from PND 22 also found a delay in preputial separation, reduction in serum testosterone and luteinizing hormone along with decreases in body, prostate, and seminal vesicle weight [82]. A third study was conducted that followed a similar dosing regimen in which results revealed a significant decrease in body weight at 200 mg/kg along with an increase in relative testes weight at 100 and 200 mg/kg. Regular testes weight was decreased at 50 and 200 mg/kg. In addition, a significant decrease in serum testosterone was noted in all atrazine concentrations, along with a corresponding increase in estradiol concentration in the 100 and 200 mg/kg treatment groups [78]. Lastly, a study exposing Wistar rodents from PND 23–53/54 found a significant reduction in body weight, ventral prostate weight, and seminal vesicle weight, but no alterations in testicular weight. Furthermore, a delay in preputial separation was once again observed [83]. Overall the results from these studies support that peripubertal atrazine exposure elicits a delay in preputial separation, decreases testosterone, and increases estradiol.

### 3.3. Adulthood Atrazine Exposure in Males

As previous studies have aimed to address the gestational and developmental aspects of atrazine toxicity, it is of equal importance to address effects during adulthood. Adult male Wistar rodents (120 days old) were exposed to 50 (15 days), 200 (15 or 40 days), or 300 (7 days) mg/kg/day atrazine. Results from this study demonstrated that atrazine exposure of 200 mg/kg for 15 days and 300 mg/kg for 7 days caused a decrease in body weight and an initial increase in relative and absolute testes and adrenal weight; while the 200 mg/kg dose for 40 days caused a reduction in relative and absolute testes weight. Furthermore, a reduction in plasma testosterone was observed in all treatments except in the 50 mg/kg dose for 15 days. The treatment of 200 mg/kg for 15 days also increased plasma and testicular estradiol. Testis morphology revealed atrazine exposure caused dilation of the seminiferous tubules at 200 mg/kg for 15 days and 300 mg/kg for 7 days. Leydig cells also showed alterations caused by atrazine exposure including irregular nuclei with an increased amount of heterochromatin [88]. It is in agreement throughout the literature that atrazine exposure decreases body weight [82,83,87]; however, alterations in testes weight has been controversial [83,87]. The results from this study showed an initial increase in testicular weight with shorter atrazine exposures, but observed a decrease with longer exposure periods. The initial increase in testes weight corresponded with the observed larger lumen of seminiferous tubules which could be caused by fluid accumulation, while the later reduction corresponded to the observed increase in testicular atrophy [88].

An additional study addressing the hormonal and histological alterations caused by atrazine, exposed adult male Sprague-Dawley rats to 38.5, 77, or 154 mg/kg/day for 30 days. Once dosing was complete, testes were removed and analyzed. A decrease in testes weight was observed in the 154 mg/kg treatment with no alterations in seminal vesicle, prostate, or epididymis weight. Histological results showed irregular and disordered arrangement of seminiferous epithelium at 154 mg/kg along with a decrease in the number of spermatozoa and an increase in spermatozoa abnormalities. A decrease in testicular markers was also observed in the 74 and 154 mg/kg treatment groups including testicular acid phosphatase (ACP), alkaline phosphatase (AKP), lactate dehydrogenase (LDH), and succinate dehydrogenase (SDH). Regarding serum hormone levels, a decrease in testosterone was observed at 74 and 154 mg/kg along with an increase in LH and FSH [84]. Histological results of the testes tissue revealed loose intercellular connections which could also disturb the junctions between Sertoli and germ cells leading to a reduction in testosterone. This reduction in testosterone levels could be responsible for the observed reduction in INH-B synthesis in the Sertoli cells. INH-B is a marker of Sertoli cell function and spermatogenesis. Additionally, the secretion of FSH and LH is regulated by testosterone and INH-B through a classic negative feedback loop. Therefore, atrazine exposure is hypothesized to damage Leydig and Sertoli cells which then causes a decrease in INH-B from Sertoli cells and a decrease in testosterone from Leydig cells. This decrease in testosterone would then cause an increase in LH and FSH. However, the majority of studies (discussed in Section 2.3) indicate that atrazine decreases LH and FSH which is contradictory to the results of this study. These inconsistent findings could be due to the differences in treatment levels and/or exposure periods. An additional study showed that adult male Wistar rats exposed to 120 or 200 mg/kg/day for 7 or 16 days showed a decrease in testicular sperm count, epididymal sperm count, and sperm motility along with an increase in number of dead and abnormal sperm [87]. It is well accepted that a decrease in sperm count combined with an increase in sperm abnormalities can increase the risk of infertility. The association between atrazine exposure and poor semen quality was also reported in an epidemiological study [91].

Literature regarding atrazine and its effects on male steroidogenesis has focused on morphological, hormonal, and proteome alterations in various rodent models. The hormone profiles and testes proteomes of adult male Wistar rats were characterized following a 3 day atrazine exposure of 5, 25, 75, or 200 mg/kg/day. Results from this study showed a significant increase in progesterone and corticosterone in the 25 and 200 mg/kg treatment groups. Additionally, an increase in luteinizing hormone and testosterone was observed only at the 25 mg/kg treatment group. Proteomic analysis of testes tissue revealed alterations in six proteins primarily correlated with testosterone. Although no steroidogenic proteins were found to be altered in this study, the majority of the proteins were linked to ubiquitin-C (UBC). UBC is a component of the ubiquitin-proteasome pathway which is part of the glucocorticoid receptor degradation response. However, progesterone receptors are also targeted for degradation in the same manner [86].

### 3.4. Developmental Origins of Atrazine Toxicity

Studies investigating the effects of early life exposure to environmental stressors or stimuli increased dramatically over the past decade. These studies seek to investigate the developmental origins of health and adult disease (DOHaD) hypothesis which states that exposure to stressors during sensitive times of an organism’s life, specifically during developmental stages, can cause changes to the genome and epigenome thereby resulting in an increased susceptibility to the development of health issues or diseases later in life [91,92]. Further complicating the establishment of a link between exposure and a disease state is the time that elapses between exposure and outward response or development of a disease [92,93]. Thus, it may take years for an individual to present a disease or dysfunctional state [94].

A few studies have started to investigate atrazine toxicity using the DOHaD exposure paradigm with rodent models. In one study, pregnant Long-Evans rats were exposed to 100 mg/kg/day atrazine from GD 15–19. Later in life observations at PND 120 revealed an increase in lateral prostate weight and inflammation in male offspring, but no alterations in serum testosterone, estrogen, or prolactin were observed. By PND 220, increased prostate weight was still observed; however, inflammation was no longer present. Furthermore, an increase in the weight of the anterior pituitary was observed. At this later time point, a decrease in prolactin was observed. Results from this study showed that *in utero* exposure combined with nursing from an atrazine treated dam enhanced the effects on prostate and pituitary weight and prostate inflammation [90]. This study is in agreement with a previous study indicating lateral prostate inflammation at PND 120 [95]. However, this could be a transient effect or require longer or higher levels of atrazine exposure to continue inflammation as it was no longer present at PND 220. Male offspring also had a delay in puberty (discussed in Section 3.1 and Section 3.2); a stated hypothesis is that the observed effects could be due to early hormonal alterations or early postnatal brain development, especially in the area of the median eminence, which is critical for the timing of preputial separation [90]. Furthermore, atrazine may effect factors in the median eminence that regulate LHRH release, levels of ghrelin (a negative regulator of LH), or alterations in leptin [96].

A second study examining the later in life effects of a gestational atrazine exposure treated pregnant Wistar rats from GD 6–21 with 1, 5, 25, or 125 mg/kg/day atrazine with evaluation of male offspring at PND 70 and PND 170. Results indicated a decrease in pituitary weight in the 125 mg/kg/day treatment group at PND 70. While analysis of testes tissue revealed no alterations in spermatid counts in testes or spermatozoa counts in the epididymides at PND 70 and 170, an increase in the number of abnormal sperm was significantly increased in the 125 mg/kg/day treatment group at PND 70 and in the 25 mg/kg/day treatment group on PND 170. Plasma testosterone levels were also unaffected at PND 70 or 170 [89]. In addition, lactational exposure in offspring from PND 4-PND 21 had a reduction in absolute testes and epididymis weights at PND 70. Evaluation at PND 170 showed a reduction in testes weight and abnormal sperm in the 125 mg/kg/day treatment group. No alterations in plasma testosterone levels were observed at any time point or dose [89]. Results from this study showed that atrazine had a minimal effect on male reproductive endpoints. This study is in contrast to the previously mentioned study [82] in which peripubertal atrazine exposure caused a reduction in plasma and intratesticular testosterone as well as reductions in seminal vesicle and prostate weights. These differences may be contributed to the differences in dosing and observational periods. In contrast to the previously mentioned studies, one study was conducted that utilized a similar experimental design in which pregnant female Sprague-Dawley rats were exposed to 1, 5, 20, or 100 mg/kg atrazine from GD 14–21. Analysis of male offspring exposed *in utero* to 1–20 mg/kg/day did not have altered testosterone production, timing of puberty, or play behavior [72].

### 3.5. Cellular and Genetic Mechanisms of Reproductive Dysfunction in Males

Steroidogenesis in males occurs in Leydig cells and is primarily mediated through the interaction of LH with the luteinizing hormone receptor (LHR). Steroidogenesis is mediated through multiple signaling pathways, including cAMP dependent regulatory events [70,74,79,80,97,98]. Numerous genes are involved in steroidogenesis and are under investigation in order to define the genetic mechanisms behind the observed reproductive dysfunction (Figure 1).

Pogrmic *et al.*, obtained Leydig cells from peripubertal rats that were treated with 50 or 200 mg/kg/day from PND 23–50. Extracellular levels of cAMP along with mRNA transcripts of luteinizing hormone receptor (*Lhr*), scavenger receptor B-1 (*Sr-b1*), steroidogenic factor-1 (*Sf-1*), steroidogenic acute regulatory protein (*Star*), 3β*-*hydroxysteroid dehydrogenase (*Hsd*), *Cyp17a1*, translocator protein (*Tspo*) and *17*β*-Hsd* were assessed. Results report that atrazine decreased cAMP, and the expression of *Lhr*, *Sr-b1*, *Star*, *Sf-1*, *Pdeb4*, *Cyp17a1*, *Tspo*, and *17*β*-Hsd*. This strong inhibition of steroidogenic genes and cAMP are mechanistic of the observed decrease in androgen production. It has been identified that the cAMP/PKA pathway is an upstream regulator of numerous steroidogenic genes and results from this study confirm this relationship. Furthermore, atrazine did not have an effect on the expression of *3*β*-Hsd*. This provides evidence that atrazine does not affect the conversion of pregnenolone to progesterone [80]. A second study conducted by the same group used short term atrazine exposure of 24 h in peripubertal rats (PND 51). Contrary to the previous study [80], an acute 24 h atrazine exposure elicited an up regulation of cAMP signaling and increased expression of steroidogenic genes (*Sf-1, Star, 17*β*-Hsd, Cyp17a1*) in Leydig cells. Confirmation by *in vivo* studies also demonstrated a stimulatory effect of atrazine on cAMP signaling and steroidogenesis [79]. These two studies combined suggest that atrazine has the potential to elicit a biphasic response through stimulatory actions following acute exposure, while chronic atrazine exposure elicits strong steroidogenic inhibition [79].

As previously stated cAMP plays a strong regulatory role and can elicit a robust increase in steroidogenesis. Key inhibitors of cAMP are phosphodiesterases (PDEs) which cause a decrease in the conversion of cAMP to 5′-AMP [70,71]. Atrazine has been identified as an inhibitor of PDEs; therefore, providing a means of the observed increase in cAMP following acute atrazine exposure [70]. A transcriptomic analysis of BLTK1 murine Leydig cells revealed an up regulation of *Pde10*, *Pde4b*, and *Pde4dip* in response to a 48 h atrazine exposure [74]. This upregulation of PDEs could be a compensatory response as a means to decrease cAMP levels; which previously were shown to be increased following short term atrazine exposure [70].

### 3.6. Tissue Levels of Atrazine in Males

As previously stated, studies examining tissue distribution in animal models is currently limited, especially in regards to male models. Thus far, a study conducted by Ross *et al.*, exposed male C57BL/6 mice to a single dose of 5, 25, 125, or 250 mg/kg atrazine and measured atrazine and metabolite levels in urine, plasma, and various tissues (liver, kidney, brain, spleen, and thymus). Urine analysis reported a dose dependent increase in atrazine and its metabolites with the highest concentration occurring in DACT which was two orders of magnitude higher than atrazine, 24 h after exposure. Atrazine and DACT were also identified in the plasma, brain, liver, kidney, spleen, and thymus tissue [99]. Although this study demonstrates that atrazine is taken up into numerous tissues, its weakness lies in the absence of testes analysis. As steroidogenesis occurs within the gonads, understanding how much atrazine was taken up and how quickly it is eliminated may aid in the understanding of the morphological, hormonal, and cellular mechanisms of atrazine toxicity. A study conducted by Fraites *et al.*, exposed pregnant Sprague-Dawley dams to 5 or 25 mg/kg/day from GD 14–20 or GD 18–20. Analysis of the male fetus (GD 20) reported a dose-dependent increase in atrazine. The male offspring (PND 10) reported high levels of DACT in the plasma while the brain contained lower and more variable levels of atrazine. Testes analysis displayed a dose-related increase in DACT between the 5 and 25 mg/kg treatment group [72]. While analysis regarding atrazine accumulation in the testes is limited, this data is a stepping stone in linking gestational atrazine exposure to previously observed morphological alterations [76,77].

### 3.7. Conclusions

Overall literature regarding the effects of atrazine on the mammalian male reproductive system report congruent findings that support the hypothesis that atrazine adversely affects the HPG axis contributing to reproductive dysfunction, but questions still remain regarding the later-in-life effects and exposure to lower atrazine doses.

## 4. The Effects of Atrazine on Reproductive Function in Anuran and Fish Models

### 4.1. The Effects of Atrazine on Reproductive Function in Anuran Models

Studies utilizing *Xenopus laevis* for examining atrazine toxicity have been under investigation for many years and have used various exposure periods and concentrations (Table 3). In a series of studies by Hayes *et al.*, atrazine exposures of 0.1–200 μg/L were reported to elicit hermaphroditism, decreases in testosterone, decreases in testosterone dependent morphogenesis, and decreased mating competition [100,101,102]. While a separate study found that atrazine at 100, 450, and 800 μg/L delayed metamorphosis of *X. laevis* [103]. Additional developmental studies with *X. laevis* report contrasting results in which exposures ranging from 0.1 to 100 μg/L did not elicit alterations in growth or larval development, sex ratios, or gonadal histology [104,105,106,107,108,109]. Other species of frogs have also been used to examine atrazine toxicity; however, similar to the *X. laevis* studies, conflicting results are reported [103,110,111].

Studies aiming to address atrazine toxicity with adult *X. laevis* show minimal alterations. A recent study exposed adult *X. laevis* to 100 μg/L atrazine for 120 days. The results obtained from this study were a delay in growth, but no hermaphroditism was reported in males. This exposure regime also caused a delay or prevented the development of seminiferous tubules along with proteomic significance in proteins associated with tight junctions and apoptosis [112]. An additional adult study with exposures from 1 to 250 μg/L also report no alterations in gonadosomatic index (GSI), germ cell morphology, or alterations in estradiol concentrations [113]. Although there are contrasting reports regarding the toxicity to *X. laevis*, it is important to take into consideration atrazine concentrations and exposure periods for future assessment.

### 4.2. The Effects of Atrazine on Reproductive Function in Fish Models

Various fish species have also been used to investigate the adverse effects of atrazine exposure. As previously stated, *in utero* and developmental periods are susceptible to chemical insults and have the potential to alter the genetic profile of developing organisms. A developmental study in which zebrafish embryos were exposed to 0, 0.3, 3, or 30 μg/L atrazine revealed a significant increase in head length following an embryonic atrazine treatment through 72 h post fertilization (hpf). Furthermore, the transcriptomic profile identified gene alterations associated with neuroendocrine and reproductive function, cell cycle, and carcinogenesis [114]. As the majority of studies focus on morphological alterations during developmental exposures, this study was unique in providing a genetic profile that could be connected to physiological changes and responses as a result of the developmental atrazine exposure [114].

**Table 3:** Reproductive effects of atrazine in anuran and fish models.

Reference	Exposure (μg/L)	Duration	Outcomes
**African Clawed Frog (*Xenopus laevis*)**			
Chen *et al.* [109]	0 or 100	Stage 46/47-120 days	No signs of hermaphroditism; Delay of seminiferous tubules; Alterations in 143 and 121 proteins in testes and ovaries, respectively; Proteins involved in cellular and metabolic processes along with apoptosis and tight junctions were altered
Hayes *et al.* [102]	0 or 2.5	Hatching-Metamorphosis (stage 66)	Decrease in plasma testosterone; Decrease in size of dermal breeding glands; Decrease in relative number of testicular tubules with mature sperm bundles which disappeared 3 years after metamorphosis; Decrease in mating competition
Kloas *et al.* [107]	0, 0.01, 0.1, 1, 25, or 100	8 dpf–metamorphosis (83 dpf)	No effect on growth or larval development; No effects observed on sex ratios or on gonadal histology
DuPreez *et al.* [106]	0, 1, 10, or 25	96 hpf–2 ypm	No effect of clutch size, hatching rate, or time to metamorphosis in F1 generation; No alterations in sex ratio of offspring (F2); No alterations in testicular morphology (F1 or F2)
Oka *et al.* [108]	0, 0.1, 1, 10, or 100	Tadpole (stage 49)–metamorphosis (stage 66)	No effect on metamorphosis, gonad development, or aromatase expression; No alterations in levels of vitellogenin
Hayes *et al.* [101]	0, 0.1, 0.4, 0.8, 1, or 25	Larvae-stage 66	Gonadal malformations; Increase in number of hermaphrodites
Coady *et al.* [105]	0, 0.1, 1, 10, or 25	72 hph-2-3 mpm	Concentrations of 0.1 and 25 μg/L did not alter mortality, metamorphosis, gonad development, or aromatase activity; 1 μg/L elicited a decrease in estradiol
Hecker *et al.* [112]	0, 10, or 100	49 days (Adult)	No alterations in germ cell development, aromatase, or plasma hormone concentrations
Hecker *et al.* [113]	0, 1, 25, or 250	36 days (Adult)	No alterations in testicular aromatase activity, expression of *cyp19*, gonadosomatic index, or plasma estradiol; A significant decrease in plasma testosterone was observed at 250 μg/L
Freeman *et al.* [103]	0, 100, 450, or 800	Stage 47 or 54–1, 2, 3, 4, or 5 weeks	Delayed metamorphosis at 100, 450, and 800 μg/L
Carr *et al.* [104]	0, 1, 10, or 25	48 hph–metamorphosis (stage 66)	No alterations in mortality, larval growth, metamorphosis, or sex ratios
Hayes *et al.* [100]	0, 0.1–200	Hatching (stage 48)–trail reabsorption (stage 66)	Hermaphroditism in males; Decrease in testosterone levels at 25 μg/L
**Leopard Frog (*Rana pipiens*)**			
Hayes *et al.* [110]	0, 0.1, or 25	2 dph–tail reabsorption (stage 66)	Underdeveloped testes; Sex reversal in some males (testicular oocytes observed)
**Spotted Marsh Frog (*Limnodynastes tasmaniensis*)**			
Spolyarich *et al.* [111]	0, 0.1, 1, 3, or 30	Gosner stage 28–44	No alterations in tadpole growth, development, or sex ratios; Testicular oocytes observed in one fish at 3 μg/L atrazine, although not statistically significant
**Fathead Minnow (*Pimephales promelas*)**			
Tillitt *et al.* [115]	0, 0.5, 5, or 50	Adult exposure (14 or 30 days)	Decrease in total egg production; Reduction in total number of spawning events; No alterations in steroid hormone levels; Testicular oocytes found in the 5 μg/Ltreatment group; Ovaries with lipid accumulation and atretic follicles observed
Battelle [116]	0, 25, or 250	Adult exposure (21 days)	No alterations in GSI, mean egg production or spawning events; No histological alterations (no atretic follicles; testicular atrophy)
Bringolf *et al.* [117]	0, 5, or 50	Adult exposure (21 days)	No alterations in survival, spawning, egg production, relative gonad weight, or in gonad histology
**Goldfish (*Carassius auratus*)**			
Nadzialek *et al.* [118]	0, 100, or 1000	Adult exposure (56 days)	No alterations in GSI, plasma concentrations of estradiol, or alterations in *cyp19a1*; Decrease in 11-ketotestosterone after 56 day exposure in the 1000 μg/L treatment
Spanò *et al.* [119]	0, 100, or 1000	Adult exposure (21 days)	No alterations in GSI; Decrease in testosterone and 11-ketotestosterone and an increase in estradiol in the 1000 μg/L treatment group; Structural disruption in testis tissue (1000 μg/L); Follicular atresia in ovary tissue in both treatments; No changes in vitellogenin
**Japanese Medaka (*Oryzias latipes*)**			
Papoulias *et al.* [120]	0, 0.5, 5, or 50	Adult (14 or 38 days)	Decrease in total egg production in all atrazine treatments after day 25 of exposure; No alterations in spawning events, GSI, aromatase protein, or whole body estradiol or testosterone
**Guppies (*Poecilia reticulate*)**			
Shenoy [121]	0, 1, or 13.5	Adult (through gestation)	Atrazine exposed male offspring were less likely to perform mating behaviors (frequency of gonopodium swings and number of forced copulatory events), and performed less frequently than control male
Shenoy [122]	0, 1, or 15	Adult male (16 weeks)	No change in number of mating attempts; Decrease in number of mating displays; Decrease in number of orange spots
**Barramundi (*Lates calcarifer*)**			
Kroon *et al.* [123]	0, 0.1, 0.5, 5, 50, or 100	Juvenile (48 h)	No alterations in *cyp19b1* in brain tissue; No alterations in VGT; Increase in plasma testosterone at 0.1 and 5 μg/L; Increase in plasma estradiol at 5 ppb
**Zebrafish (*Danio rerio*)**			
Freeman *et al.* [124]	0, 0.3, 3, or 30	1–72 hpf	Decrease in spawning events at 30 μg/L treatment; Egg bound and increase in atretic follicles in adult females exposed to 30 μg/L; Increase in ovarian progesterone in adult females exposed to 3 or 30 μg/L; Transcriptomic analysis revealed alterations in genes involved in steroidogenesis in adult female ovary
Wirbisky *et al.* [125]	0, 0.3, 3, or 30	1–72 hpf	Decrease in serotonin metabolite 5-hydroxyindoleacetic acid and serotonin turnover in adult female brain; Transcriptomic analysis revealed alterations in genes throughout the serotonergic pathway in adult female brain
Weber *et al.* [114]	0, 0.3, 3, or 30	1–72 hpf	Significant increase in head length at 72 hpf in all atrazine treatments; Transcriptomic data included genes altered in neuroendocrine and reproductive system function, cell cycle, and carcinogenesis
Corvi *et al.* [126]	0, 0.1, 1, or 10 μM	17 dpf–130 dpf	No alterations in sex ratio; No alterations in gonad development
Suzawa and Ingraham [127]	0, 0.1, 1, or 10 μM	17 dpf–6 mpf	Increase in expression of *cyp19a1* in all concentrations; Skewed sex ratio (increased number of females); Activation of MAPK pathway, SF-1, and PI3K pathway; Phosphorylation of Akt/PKB; Increase in cAMP in JEG3 cells; Upregulation in *StAR* and *cyp11a1*
Kazeto *et al.* [128]	4.6, 46, or 460 nM	17 dpf–20 dpf	No alterations in *cyp19a1* or *cyp19a2* at 4.6, 4.6, or 460 nM atrazine

Abbreviations: dpf = days post fertilization; dph = days post hatch; GSI = gonadosomatic index; hph = hours post hatch; mpm = months post metamorphosis; ppb = parts per billion (μg/L); VGT = vitellogenin; ypm = years post metamorphosis.

Multiple studies have been conducted to assess the effect of atrazine on the HPG axis in adult fish. Initial studies assessing proper reproductive function have evaluated body and testes weight, GSI, mean egg production, spawning events, and breeding behavior. A study which exposed fathead minnows to 0, 0.5, 5, or 50 μg/L during adulthood found that atrazine exposure decreased mean egg production and spawning events in all atrazine treatments [115]. In an additional study by the same group using Japanese medaka, a decrease in mean egg production was also observed at 0.5, 5, and 50 μg/L; however, no decreases in spawning events occurred [120]. Furthermore, a study exposing zebrafish embryos to atrazine concentrations of 0, 0.3, 3, or 30 μg/L from 1 to 72 hpf (only through embryogenesis) also displayed a decrease in spawning events; however, no decrease in mean egg production was observed [124]. Two contrasting studies have been reported in which no alterations in mean egg production or spawning events occurred in fathead minnows exposed to atrazine for 21 days. Battelle administered an atrazine treatment of 25 or 250 μg/L [116]; while Bringolf *et al.*, utilized an atrazine treatment of 5 or 50 μg/L [117]. One difference that may account for the discrepancy is the use of a flow-through system verses a static-renewal system. This could potentially alter atrazine uptake and kinetics in the fish. A second contributing factor could be the difference in exposure periods. The studies conducted by Battelle and Bringolf *et al.*, followed the test exposure period of 21 days as prescribed by the U.S. EPA [116,117], whereas a 38 day exposure period as used by Tillitt *et al.*, and Papoulias *et al.*, encompass a larger percent of the spawning period and may explain differences in findings [115,120].

Reductions in spawning events could be attributed to either a male or female specific effect on reproductive behavior. Although direct breeding behavior was not assessed in these studies, previous studies note alterations in reproductive behavior in various fish species [121,122,129]. Furthermore, histological alterations within gonad tissue can also contribute to the lack of spawning events. In females, atrazine was shown to cause an increase in atretic follicles [115,119,124]. The alterations in ovary histology have also been observed in pig and rodent models as previously discussed [47,48,59]. Histological analysis of male testes tissue in anuran and fish species has not revealed significant alterations as compared to adult rodent studies [84,88]. A few studies do report the occurrence of testicular oocytes [115,126]; however, the prevalence of this event is rare and was not statistically significant.

As the proper functioning of the neuroendocrine system is dependent upon hormonal regulation; numerous hormones have also been analyzed in various fish models in order to complement rodent studies. As previously discussed in rodent models, atrazine was shown to decrease GnRH, LH, FSH, and T, while increasing E_2_, P4, and PRL. In aquatic species, one of the major differences within the neuroendocrine system is that fish require 11-ketotestosterone as their main form of testosterone. A study conducted by Spanò *et al.*, reported an adult exposure of 21 days to 0, 100, or 1000 μg/L caused an increase in plasma estradiol with corresponding decreases in 11-ketotestosterone and testosterone [119]. A second study with the same atrazine concentrations, but with a 56 day exposure found no alterations in estradiol, but a significant decrease in testosterone in the 1000 μg/L treatment group [118]. Meanwhile, no alterations in steroid hormone concentrations after exposure to 0, 0.5, 5, or 50 μg/L atrazine in Japanese medaka were observed [118]. A study conducted with juvenile barramundi observed a non-linear dose response with an increase in testosterone at 0.1 and 5 ppb and no changes at 0.5, 50, or 100 μg/L. Additionally, an increase in estradiol at 5 ppb atrazine was reported, with no changes at 0.1, 0.5, 50, or 100 μg/L. However, a stable testosterone/estradiol ratio was observed [123]. A DOHaD study which exposed zebrafish embryos to 0, 0.3, 3, or 30 μg/L atrazine from 1 to 72 hpf did not elicit significant alterations in ovarian tissue levels of estradiol in adult female zebrafish. However, a significant increase in ovarian progesterone levels in the 3 and 30 μg/L atrazine treatment groups was observed [124]. This increase in progesterone is in agreement with previously mentioned rodent studies [41,44,67]. As previously noted, atrazine exposure was shown to decrease LH and FSH leading to reproductive dysfunction [46,50]. Furthermore, increases in progesterone may contribute to the inhibition of the pre-ovulatory surge of LH from the pituitary enhancing reproductive dysfunction. The results from these and previously mentioned studies show that atrazine elicits an increase in progesterone levels which can play a role in altered gonad morphology.

### 4.3. Cellular and Genetic Mechanisms of Reproductive Dysfunction in Anuran and Fish Models

Numerous cellular and genetic mechanisms behind the observed hormonal alterations caused by atrazine exposure are identified in female and male mammalian *in vitro* and *in vivo* studies (discussed in Section 2.5 and Section 3.5). Genetic studies conducted in anuran and fish species have generally focused on identifying alterations in aromatase (*CYP19A1*). Aromatase is responsible for catalyzing the conversion of testosterone to estradiol as well as androstenedione to estrone in the steroidogenic pathway (Figure 1). Research has focused on this potential mechanism since atrazine was shown not to be an estrogenic compound (*i.e*., does not bind to estrogen receptors). Current literature regarding these effects is under scrutiny as conflicting data are reported. In 2002 and 2010, Hayes *et al.* [100,102] reported that atrazine exposure resulted in hermaphroditic, demasculinized, and chemically castrated *X. laevis* at concentrations as low as 0.1 and 2.5 μg/L. Hayes hypothesized that the observed morphological alterations were due to an increase in *cyp19a1* expression which would therefore lead to an increase in estrogen and a decrease in testosterone. In addition, a study exposing zebrafish embryos to 0.3, 3, or 30 μg/L atrazine only through embryogenesis found a significant increase in *cyp19a1* in adult female ovary [114]. Although significant alterations in gene expression were observed, no alterations in ovarian tissue estradiol were reported. An additional study conducted by Suzawa and Ingraham (2008) which utilized zebrafish also identified a significant increase in *cyp19a1* and an increase in the number of female fish following atrazine exposure [127]. However, skepticism comes from these results due to the lack of information provided including the absence of the strain of zebrafish used, sample size, and mortality rates. Other contrasting studies have not demonstrated that atrazine causes alterations in *cyp19a1* expression in *X. laevis* and other aquatic species [105,106,112,113,118,123].

## 5. Epidemiological Studies with Atrazine

Epidemiological studies aiming to address the potential adverse health outcomes associated with atrazine exposure are gaining interest (Table 4). While occupational exposures typically occur due to manufacturing or field application, environmental exposures may arise from residual spray drift or most commonly from the consumption of contaminated drinking water. The aforementioned animal studies have shown the reproductive toxicity of atrazine in various animal models with varying exposure periods, treatments, and observational endpoints (Table 1, Table 2 and Table 3). Despite our knowledge; understanding the adverse reproductive effects in humans is still under investigation.

**Table 4:** Epidemiological studies addressing reproductive alterations associated with atrazine.

Reference	Results
Chevrier *et al.* [27]	Quantified levels of atrazine and/or atrazine mercapturate in urine of 5.5% of 579 pregnant females. These levels were associated with fetal growth restriction and small head circumference. No association with congenital malformations.
Cragin *et al.* [130]	Study compared females in Illinois and Vermont and found women who lived in Illinois had a higher rate of menstrual cycle irregularity, longer follicular phases, and decreased levels of menstrual cycle biomarker.
Bakke *et al.* [131]	Urine samples collected from farm families in Iowa (United States) found higher levels of urinary atrazine in farmers as compared to controls. High levels of atrazine correlated to recent field application.
Ochoa-Acuña *et al.* [18]	Study revealed higher atrazine levels in drinking water during the third trimester and entire pregnancy was associated with babies born small for their gestational age.
Winchester *et al.* [132]	Elevated levels of pesticide exposure (atrazine) from April to July were associated with higher rates of birth defects.
Curwin *et al.* [133]	Urine samples collected from farm and non-farm families in Iowa (United States) found higher levels of urinary atrazine in farm families.
Barr *et al.* [17]	Urine samples collected from individuals in Georgia (United States). Atrazine, atrazine mercapturate, and other atrazine metabolites (DACT, DEA, DIA) were found in urine samples with DACT being the most prevalent.
Mattix *et al.* [134]	Prevalence of abdominal wall defects was higher in Indiana (United States) as compared to the national rate. Comparison demonstrated a positive correlation between abdominal wall defects and atrazine exposure.
Munger *et al.* [135]	Iowa study found greater risk of intrauterine growth retardation with higher levels of atrazine in drinking water.
Villanova *et al.* [136]	Atrazine levels in water from the district of Finistère in West Brittany, France, were not associated with lower birth weight or babies born small for gestational age. Results suggest association with prematurity.

One of the most recent studies conducted by Chevrier *et al.*, examined the relationship between adverse birth outcomes and urinary biomarkers of prenatal atrazine exposure. This study was based on the PELAGIE (Perturbateurs endocriniens: Étude Longitudinale sure les Anomalies de la Grossesse, l’Infertilité et l’Enfance) cohort in the Brittany region of France. Results from this study found quantifiable levels of atrazine or atrazine mercapturate in maternal urine samples The presence of atrazine was associated with fetal growth impairment and small head circumference [27]. An additional study conducted by Bakke *et al.*, examining Iowa farmers found urinary levels of atrazine mercapturate were associated with the amount of atrazine applied to crops [131]. A second Iowa study also found higher levels of urinary atrazine in farm families verses non-farming families [133]. An additional study by Barr *et al.*, found high levels of atrazine mercapturate and its metabolites (primarily diaminochlorotriazine (DACT)) present in the urine of turf applicators and non-occupationally exposed individuals [17]. Although this study had a limited sample size, it concluded that exposure to atrazine through its application generates higher levels of atrazine mercapturate (or other atrazine metabolites) compared to low dose exposure caused by the ingestion of food and/or water.

Further studies have also shown a relationship between maternal atrazine exposure (based upon assumed drinking water levels of atrazine) and an increased risk of babies born small for their gestational age (SGA), intrauterine growth retardation (IUGR), and birth defects [18,130,131,132,134,135]. In contrast, Villanueva *et al.*, found no association between atrazine concentrations in drinking water and prevalence of SGA, prematurity, or low birth weight [136]. Observing how atrazine affects the menstrual cycle in women is also of importance due to atrazine’s ability to alter reproductive hormones (discussed in Section 2.3). A study conducted by Cragin *et al.*, compared menstrual cycles of women who lived in Illinois (where atrazine is used extensively) and Vermont (where atrazine is used sparingly) and found that Illinois women were more likely to report menstrual cycle length irregularity, to have longer follicular phases, and to have decreased levels of menstrual cycle endocrine biomarkers such as E_1_3G (major estradiol metabolite) and Pd3G (progesterone metabolite) [130]. The formerly mentioned epidemiological studies reported various outcomes associated with atrazine exposure. While the majority of studies have drawn these conclusions with water levels of atrazine ranging from 5.9 to greater than 10 μg/L [18,21,134], further reports showed that atrazine can accumulate up to 154 μg/L [22]. These studies demonstrate that atrazine exposure can elicit adverse health effects at environmentally relevant concentrations that are continually above the MCL.

There are many challenges when interpreting epidemiological studies, in regards to the previously mentioned studies; one particular challenge is that the level of municipal, unfiltered water consumption among individuals is unknown. Pregnant women and/or families may choose bottled water over tap water for all or some portions during the studies. Additionally, participants who work outside the home or travel can also be exposed to different water sources. Furthermore, obtaining accurate assessments of exposure can be challenging, as there are large seasonal differences in the types, amounts, and frequency of pesticide application [133]. Of note, is the lack of mention of dietary exposure to atrazine, while these levels are minimal (0.046–0.286 μg/kg/day), all types of exposure are required for adequate toxicological assessment [137]. A detailed literature review of atrazine exposure and pregnancy outcomes can be found in Goodman *et al.* [138].

## 6. Conclusions

Overall, the findings from this review demonstrate that atrazine affects the HPG axis. Although differences are reported dependent on life stage of exposure and dose, common themes do emerge among the laboratory studies (Figure 2). In summary, female mammalian models have addressed the effects of atrazine at numerous life stages. Gestational and prenatal studies have demonstrated a significant delay in VO and a controversial effect on mammary gland epithelium. Adult exposure studies report robust alterations in reproductive hormones including GnRH, LH, FSH, P4, and PRL. In combination, atrazine is reported to elicit a prolonged estrous cycle and histological alterations of ovarian tissue including an increase in atretic follicles. In addition, male mammalian models have begun to be of key importance in understanding reproductive dysfunction with gestational exposure demonstrating a delay in puberty. Furthermore, prenatal and adult studies show decreased levels of testosterone and altered testicular morphology in response to atrazine exposure. In addition, numerous steroidogenic genes and upstream regulators are altered due to atrazine exposure in both granulosa and Leydig cells. Anuran and fish models are also providing insight into the reproductive dysfunction and aquatic toxicity of atrazine. Studies have observed decreases in spawning events and egg production, alterations in hormone levels and gonad histology, and genetic changes. Epidemiological studies highlight the need for understanding the reproductive consequences of atrazine exposure and its impact on rural communities as exposure is evident in areas where this herbicide is used. Over the past twenty years, studies addressing the adverse reproductive effects of atrazine have encompassed multiple animal models, treatment levels, and life stages. While there are still a number of questions that need to be answered surrounding relevancy of the laboratory findings to human health risks (e.g., comparable exposure doses and continued assessment of a safe level of exposure), the sustained heavy usage of atrazine in many countries around the globe, including the United States, results in widespread environmental exposure via drinking water contamination and signifies the need for continued focus on investigating atrazine’s effects on reproductive function.

## References

[1] Plant T.M. (2015). The hypothalamo-pituitary-gonadal axis. J. Endocrinol..

[2] Wang N., Kuang L., Han B., Li Q., Chen Y., Zhu C., Chen Y., Xia F., Cang Z., Zhu C. (2015). Follicle-stimulating hormone associates with prediabetes and diabetes in postmenopausal women. Acta Diabetol..

[3] Jin J.-M., Yang W.-X. (2014). Molecular regulation of hypothalamus-pituitary-gonads axis in males. Gene.

[4] Vadakkadath M.S., Atwood C.S. (2005). The role of hypothalamic-pituitary-gonadal hormones in the normal structure and functioning of the brain. Cell Mol. Life Sci..

[5] Roy J.R., Chakraborty S., Chakraborty T.R. (2009). Estrogen-like endocrine disrupting chemicals affecting puberty in humans—A review. Med. Sci. Monit..

[6] Swedenborg E., Rüegg J., Mäkelä S., Pongratz I. (2009). Endocrine disrupting chemicals: Mechanisms of action and involvement in metabolic disorders. J. Mol. Endocrinol..

[7] Danjou A.M., Fervers B., Boutron-Ruault M.C., Philip T., Clavel-Chapelon F., Dossus L. (2015). Estimated dietary dioxin exposure and breast cancer risk among women from the French E3N prospective cohort. Breast. Cancer. Res..

[8] Swan S.H., Kruse R.L., Liu F., Barr D.B., Drobnis E.Z., Redmon J.B., Wang C., Brazil C., Overstreet J.W. (2003). Study for Future Families Research Group. Semen quality in relation to biomarkers of pesticide exposure. Environ. Health. Perspect..

[9] Vafeiadi M., Georgiou V., Chalkiadaki G., Rantakokko P., Kiviranta H., Karachaliou M., Fthenou E., Venihaki M., Sarri K., Vassilaki M. (2015). Association of Prenatal Exposure to Persistent Organic Pollutants with Obesity and Cardiometabolic Traits in Early Childhood: The Rhea Mother-Child Cohort (Crete, Greece). Environ. Health. Perspect..

[10] Xue J., Wu Q., Sakthivel S., Pavithran P.V., Vasukutty J.R., Kannan K. (2015). Urinary levels of endocrine-disrupting chemicals, including bisphenols, bisphenol a diglycidyl ethers, benzophenones, parabens, and triclosan in obese and non-obese Indian children. Environ. Res..

[11] Hanson M.A., Gluckman P.D. (2014). Early developmental conditioning of later health and disease: Physiology or pathophysiology. Physiol. Rev..

[12] Anway M.D., Cupp A.S., Uzumcu M., Skinner M.K. (2005). Epigenetic transgenerational actions of endocrine disruptors and male fertility. Science.

[13] Anway M.D., Skinner M.K. (2006). Epigenetic transgenerational actions of endocrine disruptors. Endocrinology.

[14] Guerrero-Bosagna C., Settles M., Lucker B., Skinner M.K. (2010). Epigenetic transgenerational actions of vinclozolin on promoter regions of the sperm epigenome. PLoS ONE.

[15] Fudvoye J., Bourguignon J.-P., Parent A.-S. (2014). Endocrine Disrupting Chemicals and Human Growth Maturation: A focus on early critical windows of exposure. Vitam. Horm..

[16] Vandenberg L.N. (2012). Low-dose effects of hormones and endocrine disruptors. Vitam. Horm..

[17] Barr D.B., Panuwet P., Nguyen J.V., Udunka S., Needham L.L. (2007). Assessing exposure to atrazine and its metabolites using biomonitoring. Environ. Health. Perspect..

[18] Ochoa-Acuña H., Frankenberger J., Hahn L., Carbajo C. (2009). Drinking-water herbicide exposure in Indiana and prevalence of small-for-gestational-age and preterm delivery. Environ. Health. Perspect..

[19] Eldridge J.C., Wetzel L.T., Stevens J.T., Simpkins J.W. (1999). The mammary tumor response in triazine-treated female rats: A threshold-mediated interaction with strain and species-specific reproductive senescence. Steroids.

[20] Solomon K.R., Carr J.A., du Preez L.H., Giesy J.P., Kendall R.J., Smith E.E., van der Kraak G.J. (2008). Effects of atrazine on fish, amphibians, and aquatic reptiles: A critical review. Crit. Rev. Toxicol..

[21] Rinsky J.L., Hopenhayn C., Golla V., Browning S., Bush H.M. (2012). Atrazine exposure in public drinking water and preterm birth. Public. Health. Rep..

[22] Rohr J.R., McCoy K.A. (2010). A qualitative meta-analysis reveals consistent effects of atrazine on freshwater fish and amphibians. Environ. Health. Perspect..

[23] U.S. Environmental Protection Agency (U.S. EPA) (2002). EPA 816-F-02-013. List of Contaminants and Their MCLs.

[24] Vonberg D., Vanderborght J., Cremer N., Pütz T., Herbst M., Vereecken H. (2014). 20 years of long term atrazine monitoring in a shallow aquifer in western Germany. Water. Res..

[25] Jablonowski N.D., Schäffer A., Burauel P. (2011). Still present after all these years: Persistence plus potential toxicity raise questions about the use of atrazine. Environ. Sci. Pollut. Res..

[26] World Health Organization (2011). Guidelines for Drinking Water.

[27] Chevrier C., Limon G., Monfort C., Rouget F., Garlantezec R., Petit C., Durand G., Cordier S. (2011). Urinary biomarkers of prenatal atrazine exposure and adverse birth outcomes in the PELAGIE birth cohort. Environ. Health. Perspect..

[28] Pogrmic-Majkic K., Samardzija D., Fa S., Hrubik J., Glisic B., Kaisarevic S., Andric N. (2014). Atrazine enhances progesterone production through activation of multiple signaling pathways in FSH-stimulated rat granulosa cells: Evidence for premature luteinization. Biol. Reprod..

[29] Fa S., Pogrmic-Majkic K., Samardzija D., Glisic B., Kaisarevic S., Kovacevic R., Andric N. (2013). Involvement of ERK1/2 signaling pathway in atrazine action on FSH-stimulated LHR and CYP19A1 expression in rat granulosa cells. Toxicol. Appl. Pharmacol..

[30] Basini G., Bianchi F., Bussolati S., Baioni L., Ramoni R., Grolli S., Conti V., Bianchi F., Grasselli F. (2011). Atrazine disrupts steroidogenesis, VEGF and NO production in swine granulosa cells. Ecotoxicol. Environ. Saf..

[31] Tinfo N.S., Hotchkiss M.G., Buckalew A.R., Zorrilla L.M., Cooper R.L., Laws S.C. (2011). Understanding the effects of atrazine on steroidogenesis in rat granulosa and H295R adrenal cortical carcinoma cells. Reprod. Toxicol..

[32] Holloway A.C., Anger D.A., Crankshaw D.J., Wu M., Foster W.G. (2008). Atrazine-induced changes in aromatase activity in estrogen sensitive target tissues. J. Appl. Toxicol..

[33] Sanderson J.T., Seinen W., Giesy J.P., van den Berg M. (2000). 2-chloro-*S*-triazine herbicides induce aromatase (CYP19) activity in H295R human adrenocortical carcinoma cells: A novel mechanism for estrogenicity?. Toxicol. Sci..

[34] Davis L.K., Murr A.S., Best D.S., Fraites M.J., Zorrilla L.M., Narotsky M.G., Stoker T.E., Goldman J.M., Cooper R.L. (2011). The effects of prenatal exposure to atrazine on pubertal and postnatal reproductive indices in the female rat. Reprod. Toxicol..

[35] Hovey R.C., Coder P.S., Wolf J.C., Sielken R.L., Tisdel M.O., Breckenridge C.B. (2011). Quantitative assessment of mammary gland development in female Long Evans rats following in utero exposure to atrazine. Toxicol. Sci..

[36] Rayner J.L., Enoch R.R., Fenton S.E. (2005). Adverse effects of prenatal exposure to atrazine during a critical period of mammary gland growth. Toxicol. Sci..

[37] Rayner J.L., Wood C., Fenton S.E. (2004). Exposure parameters necessary for delayed puberty and mammary gland development in Long-Evans rats exposed in utero to atrazine. Toxicol. Appl. Pharmacol..

[38] Ashby J., Tinwell H., Stevens J., Pastoor T., Brechenridge C.B. (2002). The effects of atrazine on the sexual maturation of female rats. Regul. Toxicol. Pharmacol..

[39] Laws S.C., Ferrell J.M., Stoker T.E., Schmid J., Cooper R.L. (2000). The effects of atrazine on female Wistar rats: An evaluation of the protocol for assessing pubertal development and thyroid function. Toxicol. Sci..

[40] Foradori C.D., Sawhney C.P., Tisdel M., Yi K.D., Simpkins J.W., Handa R.J., Breckenridge C.B. (2014). The effect of atrazine administered by gavage or in diet on the LH surge and reproductive performance in intact female Sprague-Dawley and Long Evans rats. Birth Defects Res. B Dev. Reprod. Toxicol..

[41] Goldman J.M., Davis L.K., Murr A.S., Cooper R.L. (2013). Atrazine-induced elevation or attenuation of the LH surge in the ovariectomized, estrogen-primed female rat: Role of adrenal progesterone. Reproduction.

[42] Foradori C.D., Zimmerman A.D., Hinds L.R., Zuloaga K.L., Breckenridge C.B., Handa R.J. (2013). Atrazine inhibits pulsatile gonadotropin-releasing hormone (GnRH) release without altering GnRH messenger RNA or protein levels in the female rat. Biol. Reprod..

[43] Quignot N., Arnaud M., Robidel F., Lecomte A., Tournier M., Cren-Olivé C., Barouki R., Lemazurier E. (2012). Characterization of endocrine-disrupting chemicals based on hormonal balance disruption in male and female adult rats. Reprod. Toxicol..

[44] Taketa Y., Yoshida M., Inoue K., Takahashi M., Sakamoto Y., Watanabe G., Taya K., Yamate J., Nishikawa A. (2011). Differential stimulation pathways of progesterone secretion from newly formed corpora lutea in rats treated with ethylene glycol monomethyl ether, sulpiride, or atrazine. Toxicol. Sci..

[45] Foradori C.D., Hinds L.R., Hanneman W.H., Handa R.J. (2009). Effects of atrazine and its withdrawal on gonadotropin-releasing hormonee neuroendocrine function in the adult female Wistar rat. Biol. Reprod..

[46] Foradori C.D., Hinds L.R., Hanneman W.H., Legare M.E., Clay C.M., Handa R.J. (2009). Atrazine inhibits pulsatile luteinizing hormone release without altering pituitary sensitivity to a gonadotropin-releasing hormone receptor agonist in female Wistar rats. Biol. Reprod..

[47] Shibayama H., Kotera T., Shinoda Y., Hanada T., Kajihara T., Ueda M., Tamura H., Ishibashi S., Yamashita Y., Ochi S. (2009). Collaborative work on evaluation of ovarian toxicity. 14) Two- or four-week repeated-dose studies and fertility study of atrazine in female rats. Toxicol. Sci..

[48] Juliani C.C., Silva-Zacarin E.C.M., Santos D.C., Boer P.A. (2008). Effects of atrazine on female Wistar rats: Morphological alterations in ovarian follicles and immunocytochemical labeling of 90 kDa heat shock protein. Micron.

[49] McMullin T., Andersen M.E., Nagahara A., Lund T.D., Pak T., Handa R.J., Hanneman W.H. (2004). Evidence that atrazine and diaminochlorotriazine inhibit the estrogen/progesterone induced surge of luteinizing hormone in female Sprague-Dawley rats without changing estrogen receptor action. Toxicol. Sci..

[50] Cooper R.L., Stoker T.L., Tyrey L., Goldman J.M., McElroy W.K. (2000). Atrazine disrupts the hypothalamic control of pituitary-ovarian function. Toxicol. Sci..

[51] Wetzel L.T., Luempert L.G., Breckenridge C.B., Tisdel M.O., Stevens J.T., Thakur A.K., Extrom P.J., Eldridge J.C. (1994). Chronic effects of atrazine on estrus and mammary tumor formation in female Sprague-Dawley and Fischer 344 rats. J. Toxicol. Environ. Health.

[52] Stevens J.T., Breckenridge C.B., Wetzel L.T., Gillis J.H., Luempert L.G., Eldridge J.C. (1994). Hypothesis for mammary tumorigenesis in Sprague-Dawley rats exposed to certain triazine herbicides. J. Toxicol. Environ. Health..

[53] Stevens J.T., Breckenridge C.B., Wetzel L. (1999). A risk characterization for atrazine: Oncogenicity profile. J. Toxicol. Environ. Health. Part. A.

[54] Pintér A., Török G., Börzsönyi M., Surján A., Csík M., Kelecsényi Z., Kocsis Z. (1990). Long-term carcinogenicity bioassay of the herbicide atrazine in F344 rats. Neoplasma.

[55] Thakur A.K., Wetzel L.T., Voelker R.W., Wakefield A.E., Ballantine L.G., MacFarland J.E., Hackett D.S. (1998). Results of a two-year oncogenicity study in Fischer 344 rats with atrazine. Triazine Herbicides: Risk Assessment.

[56] Simpkins J.W., Swenberg J.A., Weiss N., Brusick D., Eldridge J.C., Stevens J.T., Handa R.J., Hovey R.C., Plant T.M., Pastoor T.P. (2011). Atrazine and Breast Cancer: A framework assessment of the toxicological and epidemiological evidence. Toxicol. Sci..

[57] Cooper R.L., Laws S.C., Das P.C., Narotsky M.G., Goldman J.M., Tyrey E.L., Stoker T.E. (2007). Atrazine and reproductive function: Mode and mechanism of action studies. Birth Defects Res. B Dev. Reprod Toxicol..

[58] Eldridge J.C., Wetzel L.T., Tyrey L. (1999). Estrous cycle patterns of Sprague-Dawley rats during acute and chronic atrazine administration. Reprod. Toxicol..

[59] Gojmerac T., Kniewald J. (1989). Atrazine biodegeneration in rats: A model for mammalian metabolism. Bull. Environ. Contam. Toxicol..

[60] Das P.C., McElroy W.K., Cooper R.L. (2000). Differential modulation of catecholamines by chlorotriazine herbicides in pheochromocytoma (PC12) cells *in vitro*. Toxicol. Sci..

[61] Lin Z., Dodd C.A., Xiao S., Krishna S., Ye X., Filipov N.M. (2014). Gestational and lactational exposure to atrazine via the drinking water causes specific behavioral deficits an selectively alters monoaminergic systems in C57BL/6 mouse dams, juvenile and adult offspring. Toxicol. Sci..

[62] Shafer T.J., Ward T.R., Meacham C.A., Cooper R.L. (1999). Effects of chlorotriazine herbicide, cyanazine, on GABA_A_ receptors in cortical tissue from rat brain. Toxiocology.

[63] Gottsch M.L., Cunningham M.J., Smith J.T., Popa S.M., Acohido B.V., Crowley W.F., Seminara S., Clifton D.K., Steiner R.A. (2004). A role for kisspeptins in the regulation of gonadotropin secretion in the mouse. Endocrinology.

[64] Navarro V.M., Castellano J.M., Fernández-Fernández R., Tovar S., Roa J., Mayen A., Nogueiras R., Vazquez M.J., Barreiro M.L., Magni P. (2005). Characterization of the potent luteinizing hormone-releasing activity of KiSS-1 peptide, the natural ligand of GPR54. Endocrinology.

[65] Gojmerac T., Kartal B., Ćurić S., Žurić M., Kušević S., Cventi Z. (1996). Serum biochemical changes associated with cystic ovarian degeneration in pigs after atrazine treatment. Toxicol. Lett..

[66] Hirshfield A.N. (1997). Overview of ovarian follicular development: Considerations for the toxicologist. Environ. Mol. Mutagen..

[67] Fraites M.J.P., Cooper R.L., Buckalew A., Jayaraman S., Mills L., Laws S.C. (2009). Characterization of the hypothalamic-pitutiary-adrenal axis response to atrazine and metabolites in the female rat. Toxicol. Sci..

[68] Telfer E., Gosden R.G., Faddy M.J. (1991). Impact of exogenous progesterone on ovarian follicular dynamics and function in mice. J. Reprod. Fertil..

[69] Fan H.Y., Liu Z., Shimada M., Sterneck E., Johnson P.F., Hedrick S.M., Richards J.S. (2009). MAPK3/1 (ERK1/2) in ovarian granulosa cells are essential for female fertility. Science..

[70] Kucka M., Pogrmic-Majkic K., Fa S., Stojilkovic S.S., Kovacevic R. (2012). Atrazine acts as an endocrine disrupter by inhibiting cAMP-specific phosphodiesterase-4. Toxicol. Appl. Pharmacol..

[71] Roberge M., Hakk H., Larsen G. (2004). Atrazine is a competitive inhibitor of phosphodiesterase but does not affect the estrogen receptor. Toxicol. Lett..

[72] Fraites M.J., Narotsky M.G., Best D.S., Stoker T.E., Davis L.K., Goldman J.M., Hotchkiss M.G., Klinefelter G.R., Kamel A., Qian Y. (2011). Gestational atrazine exposure: Effects on male reproductive development and metabolite distribution in the dam, fetus, and neonate. Reprod. Toxicol..

[73] Quignot N., Tournier M., Pouech C., Cren-Olive C., Barouki R., Lemazurier E. (2012). Quantification of steroids and endocrine disrupting chemicals in rat ovaries by LC-MS/MS for reproductive toxicology assessment. Anal. Bioanal. Chem..

[74] Forgacs A.L., D’Souza M.L., Huhtaniemi I.T., Rahman N.A., Zacharewski T.R. (2013). Triazine herbicides and their chlorometabolites alter steroidogenesis in BLTK1 murine Leydig cells. Toxicol. Sci..

[75] Abarikwu S.O., Farombi E.O., Kashyap M.P., Pant A.B. (2011). Atrazine induces transcriptional changes in marker genes associated with steroidgogenesis in primary cultures of rat Leydig cells. Toxicol. Vitr..

[76] Stanko J.P., Enoch R.R., Rayner J.L., Davis C.C., Wolf D.C., Malarkey D.E., Fenton S.E. (2010). Effects of prenatal exposure to a low dose atrazine metabolite mixture on pubertal timing and prostate development of male Long-Evans rats. Reprod. Toxicol..

[77] Rosenberg B.G., Chen H., Folmer J., Liu J., Papadopoulos V., Zirkin B.R. (2008). Gestational Exposure to atrazine: Effects on the postnatal development of male offspring. J. Androl..

[78] Jin Y., Wang L., Fu Z. (2013). Oral exposure to atrazine modulates hormone synthesis and the transcription of steroidogeneic genes in male peripubertal mice. Gen. Comp. Endocrinol..

[79] Pogrmic-Majkic K., Fa S., Dakic V., Kaisarevic S., Kovacevic R. (2010). Upregulation of peripubertal rat Leydig cell steroidogenesis following 24 h *in vitro* and *in vivo* exposure to atrazine. Toxicol. Sci..

[80] Pogrmic K., Fa S., Dakic V., Kaisarevic S., Kovacevic R. (2009). Atrazine oral exposure of peripubertal male rats downregulates steroidogenesis gene expression in Leydig cells. Toxcol. Sci..

[81] Friedmann A.S. (2002). Atrazine inhibition of testosterone production in rat males following peripubertal exposure. Reprod. Toxicol..

[82] Trentacoste S.V., Friedmann A.S., Youker R.T., Breckenridge C.B., Zirkein B.R. (2001). Atrazine effects on testosterone levels and androgen-dependent reproductive orangs in peripubertal male rats. J. Androl..

[83] Stoker T.E., Laws S.C., Guidici D.L., Cooper R.L. (2000). The effect of atrazine on puberty in male Wistar rats: An evaluation in the protocol for the assessment of pubertal development and thyroid function. Toxicol. Sci..

[84] Song Y., Jia Z.C., Chen J.Y., Hu J.X., Zhang L.S. (2014). Toxic effects of atrazine on reproductive system of male rats. Biomed. Environ. Sci..

[85] Jin Y., Wang L., Chen G., Lin X., Miao W., Fu Z. (2014). Exposure of mice to atrazine and its metabolite diaminochlorotriazine elicits oxidative stress and endocrine disruption. Environ. Toxciol. Pharmacol..

[86] Riffle B.W., Klinefelter G.R., Cooper R.L., Winnik W.M., Swank A., Jayaraman S., Suarez J., Best D., Laws S.C. (2014). Novel molecular events associated with altered steroidogenesis induced by exposure to atrazine in the intact and castrate male rat. Reprod. Toxicol..

[87] Abarikwu S.O., Adesiyan A.C., Oyeloja T.O., Oyeyemi M.O., Farombi E.O. (2010). Changes in sperm characteristics and induction of oxidative stress in the testis and epididymis of experimental rats by a herbicide, atrazine. Arch. Environ. Contam. Toxicol..

[88] Victor-Costa A.B., Bandeira S.M., Oliveira A.G., Mahecha G.A., Oliveira C.A. (2010). Changes in testicular morphology and steroidogenesis in adult rats exposed to Atrazine. Reprod. Toxicol..

[89] De Sesso J.M., Scialli A.R., White T.E.K., Breckenridge C.B. (2014). Multigeneration reproduction and male development toxicity studies on atrazine in rats. Birth Defects Res. B Dev. Reprod. Toxicol..

[90] Rayner J.L., Enoch R.R., Wolf D.C., Fenton S.E. (2007). Atrazine-induced reproductive tract alterations after transplancental and/or lactational exposure in male Long-Evans rats. Toxicol. Appl. Pharmacol..

[91] Barouki R., Gluckman P.D., Grandjean P., Hanson M., Heindel J.J. (2012). Developmental origins of non-communicable disease: Implications for research and public health. Environ. Health..

[92] Zhang X., Ho S.M. (2011). Epigenetics meets endocrinology. J. Mol. Endocrinol..

[93] Gluckman P.D., Hanson M.A., Cooper C., Thornburg K.L. (2008). Effect of in utero and early-life conditions on adult health and disease. N. Engl. J. Med..

[94] Jirtle R.L., Skinner M.K. (2007). Environmental epigenomics and disease susceptibility. Nat. Rev. Genet..

[95] Stoker T.E., Robinette C.L., Cooper R.L. (1999). Maternal exposure to atrazine during lactation suppresses suckling-induced prolactin release and results in prostatitis in the adult offspring. Toxicol. Sci..

[96] Ojeda S.R., Ma Y.J., Lee B.T., Prevot V. (2000). Glia-to-neuron signaling and the neuroendocrine control of female puberty. Recent. Prog. Horm. Res..

[97] Manna P.R., Chandrala S.P., Jo Y., Stocco D.M. (2006). cAMP-independent signaling regulates steroidogenesis in mouse Leydig cells in the absence of StAR phosphorylation. J. Mol. Endocrinol..

[98] Manna P.R., Jo Y., Stocco D.M. (2007). Regulation of Leydig cell steroidogenesis by extracellular signal-related kinase 1/2: Role of protein kinase A and protein kinase C signaling. J. Endocrinol..

[99] Ross M.K., Jones T.L., Filipov N.M. (2009). Disposition of the herbicide 2-chloro-4-(ethylamino)-6-(isopropylamino)-*S*-triazine (atrazine) and its major metabolites in mice: A liquid chromatography/mass spectrometry analysis of urine, plasma, and tissue levels. Drug Metab. Dispos..

[100] Hayes T.B., Collins A., Lee M., Mendoza M., Noriega N., Stuart A.A., Vonk A. (2002). Hermaphroditic, demasculinized frogs after exposure to the herbicide atrazine at low ecologically relevant doses. Proc. Natl. Acad. Sci. USA.

[101] Hayes T.B., Stuart A.A., Mendoza M., Collins A., Noriega N., Vonk A., Johnston G., Liu R., Kpodzo D. (2006). Characterization of atrazine-induced gonadal malformations in African clawed frogs (*Xenopus laevis*) and comparisons with effects of an androgen antagonist (cyproterone acetate) and exogenous estrogen (17β-estradiol): Support for the demasculinization/feminization hypothesis. Environ. Health Perspect..

[102] Hayes T.B., Khoury V., Narayan A., Nazir M., Park A., Brown T., Adame L., Chan E., Buchholz D., Stueve T. (2010). Atrazine induces complete feminization and chemical castration in male African clawed frogs (*Xenopus laevis*). Proc. Natl. Acad. Sci. USA.

[103] Freeman J.L., Rayburn A.L. (2005). Developmental impact of atrazine on metamorphing *Xenopus laevis* as revealed by nuclear analysis and morphology. Enivron. Toxicol. Chem..

[104] Carr J.A., Gentles A., Smith E.E., Goleman W.L., Urquidi L.J., Thuett K., Kendall R.J., Giesy J.P., Gross T.S., Solomon K.R. (2003). Response of larval *Xenopus laevis* to atrazine: Assessment of growth, metamorphosis, and gonadal and laryngeal morphology. Environ. Toxicol. Chem..

[105] Coady K.K., Murphy M.B., Villeneuve D.L., Hecker M., Jones P.D., Carr J.A., Solomon K.R., Smith E.E., van der Kraak G., Kendall R.J. (2005). Effects of atrazine on metamorphosis, growth, laryngeal and gonadal development, aromatase activity, and sex steroid concentrations in *Xenopus laevis*. Ecotoxicol. Environ. Saf..

[106] Du Preez L.H., Kunene N., Everson G.J., Carr J.A., Giesy J.P., Gross T.S., Hosmer A.J., Kendall R.J., Smith E.E., Solomon K.R. (2008). Reproduction, larval growth, and reproductive development in African clawed frogs (*Xenopus laevis*) exposed to atrazine. Chemosphere.

[107] Kloas W., Lutz I., Springer T., Krueger H., Wolf J., Holden L., Hosmer A. (2009). Does atrazine influence larval development and sexual differentiation in *Xenopus laevis*?. Toxicol. Sci..

[108] Oka T., Tooi O., Mitsui N., Miyahara M., Ohnishi Y., Takase M., Kashiwagi A., Shinkai T., Santo N., Iguchi T. (2008). Effect of atrazine on metamorphosis and sexual differentiation in *Xenopus laevis*. Aquat. Tox..

[109] Chen X., Wang J., Zhu H., Ding J., Peng Y. (2015). Proteomics analysis of *Xenopus laevis* gonad tissue following chronic exposure to atrazine. Environ. Toxicol. Chem..

[110] Hayes T., Haston K., Tsui M., Hoang A., Haeffele C., Vonk A. (2003). Atrazine-induced hermaphroditism at 0.1 ppb in American leopard frogs (*Rana pipiens*): Laboratory and field evidence. Environ. Health Perspect..

[111] Spolyarich N., Hyne R., Wilson S., Palmer C., Byrne M. (2010). Growth, development and sex ratios of Spotted Marsh Frog (*Limnodynastes tasmaniensis*) larvae exposed to atrazine and a herbicide mixture. Chemosphere.

[112] Hecker M., Kim W.J., Park J.W., Murphy M.B., Villeneuve D., Coady K.K., Jones P.D., Solomon K.R., van der Kraak G., Carr J.A. (2005). Plasma concentrations of estradiol and testosterone, gonadal aromatase activity and ultrastructure of the testis in *Xenopus laevis* exposed to estradiol or atrazine. Aquat. Toxicol..

[113] Hecker M., Park J.W., Murphy M.B., Jones P.D., Solomon K.R., van der Kraak G., Carr J.A., Smith E.E., du Preez L., Kendall R.J. (2005). Effects of atrazine on CYP19 gene expression and aromatase activity in testes and on plasma sex steroid concentrations of male African clawed frogs (*Xenopus laevis*). Toxicol. Sci..

[114] Weber G.J., Sepúlveda M.S., Peterson S.M., Lewis S.L., Freeman J.L. (2013). Transcriptome alterations following developmental atrazine exposure in zebrafish are associated with disruption of neuroendocrine and reproductive system function, cell cycle, and carcinogenesis. Toxicol. Sci..

[115] Tillitt D.E., Papoulias D.M., Whyte J.J., Richter C.A. (2010). Atrazine reduces reproduction in fathead minnow (*Pimephales promelas*). Aquat. Toxicol..

[116] Battelle Corporation, 2005. Multi-chemical evaluation of the short-term reproduction assay with the fathead minnow. Draft Final Report to U.S. Environmental Protection Agency.

[117] Bringolf R.B., Belden J.B. (2004). Summerfelt, R.C. Effects of atrazine on fathead minnow in a short-term reproductive assay. Environ. Toxicol. Chem..

[118] Nadzialek S., Spanò L., Mandiki S.N., Kestemont P. (2008). High doses of atrazine do not disrupt activity and expression of aromatase in female gonads of juvenile goldfish *(Carassius auratus L.*). Ecotoxicology.

[119] Spanò L., Tyler C.R., van Aerle R., Devos P., Mandiki S.N., Silvestre F., Thomé J.P., Kestemont P. (2004). Effects of atrazine on sex steroid dynamics, plasma vitellogenin concentration and gonad development in adult goldfish (*Carassius auratus*). Aquat. Toxicol..

[120] Papoulais D.M., Tillitt D.E., Talykina M.G., Whyte J.J., Richter C.A. (2014). Atrazine reduces reproduction in Japanese medaka. Aquat. Toxicol..

[121] Shenoy K. (2012). Environmentally realistic exposure to the herbicide atrazine alters some sexually selected traits in male guppies. PLoS ONE.

[122] Shenoy K. (2014). Prenatal exposure to low doses of atrazine affects mating behaviors in male guppies. Horm. Behav..

[123] Kroon F.J., Hook S.E., Jones D., Metcalfe S., Osborn H.L. (2014). Effects of atrazine on endocrinology and physiology in juvenile barramundi, *Lates calcarifer* (Bloch). Environ. Toxicol. Chem..

[124] Freeman J.L., Wirbisky S.E., Weber G.J., Sepúlveda M.S. A developmental origin of adult reproductive dysfunction in the zebrafish associated with an embryonic exposure to the herbicide atrazine. Proceedings of PPTOX IV: Environmental stressors in disease and implications for human health.

[125] Wirbisky S.E., Weber G.J., Sepúlveda M.S., Xiao C., Cannon J.R., Freeman J.L. (2015). Developmental origins of neurotransmitter and transcriptome alterations in adult female zebrafish exposed to atrazine during embryogenesis. Toxicology.

[126] Corvi M.M., Stanley K.A., Peterson T.S., Kent M.L., Feist S.W., la Du J.K., Volz D.C., Hosmer A.J., Tanguay R.L. (2012). Investigating the impact of chronic atrazine exposure on sexual development in zebrafish. Birth Defects Res. B Dev. Reprod. Toxicol..

[127] Suzawa M., Ingraham H.A. (2008). The herbicide atrazine activates endocrine gene networks via non-steroidal NR5A nuclear receptors in fish and mammalian cells. PLoS ONE.

[128] Kazeto Y., Place A.R., Trant J.M. (2004). Effects of endocrine disrupting chemicals on the expression of CYP19 genes in zebrafish (*Danio. rerio*) juveniles. Aquat. Toxicol..

[129] Moore A., Lower N. (2001). The impact of two pesticides on olfactory-mediated endocrine function in mature male Atlantic salmon (*Salmo salar L*.) parr. Comp. Biochem. Physiol. B Biochem. Mol. Biol..

[130] Cragin L.A., Kesner J.S., Bachand A.M., Barr D.B., Meadows J.W., Krieg E.F., Reif J.S. (2011). Menstrual cycle characteristics and reproductive hormone levels in women exposed to atrazine in drinking water. Environ. Res..

[131] Bakke B., de Roos A.J., Barr D.B., Stewart P.A., Blair A., Freeman L.B., Lynch C.F., Allen R.H., Alavanja M.C., Vermeulen R. (2009). Exposure to atrazine and selected non-persistent pesticides among corn farmers during a growing season. J. Exp. Sci. Environ. Epidemiol..

[132] Winchester P.D., Huskins J., Ying J. (2009). Agrichemicals in surface water and birth defects in the United States. Acta Paediatr..

[133] Curwin B.D., Hein M.J., Sanderson W.T., Striley C., Heederik D., Kromhout H., Reynolds S.J., Alavanja M.C. (2007). Urinary pesticide concentrations among children, mothers and fathers living in farm and non-farm households in Iowa. Ann. Occup. Hyg..

[134] Mattix K.D., Winchester P.D., Scherer L.R. (2007). Incidence of abdominal wall defects is related to surface water atrazine and nitrate levels. J. Pediatr. Surg..

[135] Munger R., Isacson P., Hu S., Burns T., Hanson J., Lynch C.F., Cherryholms K., van Drope P., Hausler W.J. (1997). Intrauterine growth retardation in Iowa communities with herbicide-contaminated drinking water supplies. Environ. Health Perspect..

[136] Villanueva C.M., Durand G., Coutté M.B., Chevrier C., Cordier S. (2005). Atrazine in municipal drinking water and risk of low birth weight, preterm delivery, and small-for-gestational-age status. Occup. Environ. Med..

[137] Belloni V., Dessì-Fulgheri F., Zaccaroni M., Di Consiglio E., De Angelis G., Testai E., Santochirico M., Alleva E., Santucci D. (2011). Early exposure to low doses of atrazine affects behavior in juvenile and adult CD1 mice. Toxicology..

[138] Goodman M., Mandel J.S., DeSesso J.M., Scialli A.R. (2014). Atrazine and pregnancy outcomes: A systemic review of epidemiologic evidence. Birth Defects Res. B Dev. Reprod. Toxicol..

